# Pinnatifidenyne-Derived Ethynyl Oxirane Acetogenins from *Laurencia viridis*

**DOI:** 10.3390/md16010005

**Published:** 2017-12-29

**Authors:** Adrián Morales-Amador, Caterina R. de Vera, Olivia Márquez-Fernández, Antonio Hernández Daranas, José M. Padrón, José J. Fernández, María L. Souto, Manuel Norte

**Affiliations:** 1 Instituto Universitario de Bio-Orgánica Antonio González (IUBO AG), Centro de Investigaciones Biomédicas de Canarias (CIBICAN), Universidad de La Laguna (ULL), Avenida Astrofísico Francisco Sánchez 2, 38206 Tenerife, Spain; adrian_tf@hotmail.com (A.M.-A.); caterina.rdv@gmail.com (C.R.d.V.); adaranas@ull.es (A.H.D.); jmpadron@ull.es (J.M.P.); 2 Departamento de Química Orgánica, Universidad de La Laguna (ULL), Avenida Astrofísico Francisco Sánchez s/n, 38206 Tenerife, Spain; 3 Facultad de Ciencias Biológicas-Universidad Veracruzana, Circuito Gonzalo Aguirre Beltrán s/n, Zona Universitaria C.P., Xalapa 91090, Veracruz, Mexico; mafo68@yahoo.com.mx; 4 Instituto de Productos Naturales y Agrobiología (IPNA), CSIC, Avenida Astrofísico Francisco Sánchez 2, 38206 Tenerife, Spain

**Keywords:** pinnatifidenyne, ethynyl oxiranes, C_15_ acetogenins, marine natural product, *Laurencia*, DFT calculations, antiproliferative activity

## Abstract

Red algae of *Laurencia* continue to provide wide structural diversity and complexity of halogenated C_15_ acetogenin medium-ring ethers. Here, we described the isolation of three new C_15_ acetogenins (**3**–**5**), and one truncated derivative (**6**) from *Laurencia viridis* collected on the Canary Islands. These compounds are interesting variations on the pinnatifidenyne structure that included the first examples of ethynyl oxirane derivatives (**3**–**4**). The structures were elucidated by extensive study of NMR (Nuclear Magnetic Resonance) data, *J*-based configuration analysis and DFT (Density Functional Theory) calculations. Their antiproliferative activity against six human solid tumor cell lines was evaluated.

## 1. Introduction

Medium-ring haloethers of *Laurencia*, a significant subset of biologically active marine natural products [1,2], continue to challenge innovative efforts as attractive targets for the synthesis of stereochemically rich medium-sized oxacyclic compounds [3,4] or to explore biogenetic hypothesis [5,6]. 

*Z*-Pinnatifidenyne and *E*-pinnatifidenyne (**1**–**2**) are representative and were reported in 1982 after isolation from the red algae *Laurencia pinnatifida* collected at Canary Islands [7]. Their structures were established by espectroscopic methods, X-ray diffraction analysis and chemical correlations, although, in 1991, the absolute configurations were reassigned based on a later X-ray analysis [8]. Recent synthetic approaches and biosynthetic studies of these eight-membered compounds have been reported [9,10].

**Figure 1:**
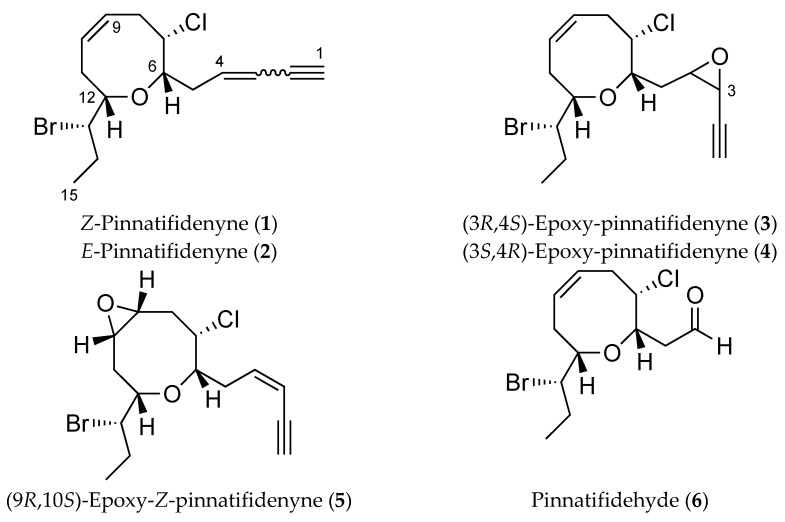
Structures of pinnatifidenynes and new metabolites.

As part of our continuing interest on the chemistry of the genus *Laurencia* [11,12,13,14] and during the course of our anticancer drug discovery program, we report the isolation of three new C_15_ acetogenins and one truncated derivative from *Laurencia viridis* (Figure 1)*.* The structures were elucidated based on detailed analysis of 1D and 2D NMR data and revealed that these compounds are variations on the pinnatifidenyne structure with biogenetic relevance. In addition, a detailed study of NMR chemical shifts by DFT calculations analysis was also undertaken and their antiproliferative activity against human cancer cell lines A549 (lung), HBL-100 (breast), HeLa (cervix), SW1573 (lung), T-47D (breast) and WiDr (colon) was evaluated.

## 2. Results and Discussion

(3*R*,4*S*)-Epoxy-pinnatifidenyne (**3**) was isolated with yellow amorphous solid appearance. A molecular formula of C_15_H_20_BrClO_2_ was deduced by the presence of three pseudomolecular [M + Na]^+^ peaks in the HR-ESI-MS (High Resolution Electro Spray Ionization Mass Spectroscopy) spectrum at *m*/*z* 369.0227, 371.0216 and 373.0201 (ratio: 76:100:24, calcd. 369.0233, 371.0203, 371.0212 and 373.0183).

The ^1^H NMR spectrum revealed the presence of signals for two olefinic protons (δ_H_ 5.93 and 5.72), six deshielded methines (δ_H_ 4.12, 3.97, 3.95, 3.65, 3.52 and 3.25), one acetylenic proton (δ_H_ 2.39), four methylenes (ranging from δ_H_ 1.64 to 2.96) and a terminal methyl group (δ_H_ 1.08) (Table 1).

According to the molecular formula, the ^13^C NMR data (Table 2) along with the analysis of the edited HSQC (Heteronuclear Single Quantum Correlation) spectrum confirmed the presence of two olefinic carbons (δ_C_ 131.0 and 128.9), six heteroatom-bearing methynes (δ_C_ 83.1, 77.5, 65.8, 61.1, 55.3 and 45.6), four methylenes (δ_C_ 34.5, 34.3, 30.0 and 27.1) and one methyl (δ_C_ 12.8). Careful examination of the homonuclear and heteronuclear NMR correlations exhibited in the COSY (Correlation SpectroscopY) and HSQC spectra allowed to observe a single ^1^H-^1^H spin system, C-3→C-15, containing a double bond between C-9 and C-10 together with heteroatoms located on carbons C-3, C-4, C-6, C-7, C-12 and C-13. Moreover, HMBC (Heteronuclear Multiple Bond Correlation) cross-peaks from H-6 (δ_H_ 4.12) to C-12 (δ_C_ 83.1) established an ether linkage between C-6 and C-12, thus indicating the presence of a 3,4,7,8-tetrahydro-2H-oxocin heterocycle. The presence of a terminal epoxy alkyne was evident by the characteristic ^1^H and ^13^C signals (*δ*_H_*/δ*_C_, 3.25, ddd, *J* = 3.8, 4.0, 8.4 Hz/55.3 (CH-4), 3.52, dd, *J* = 1.7, 4.0 Hz/45.6 (CH-3), 78.6 (C-2), and 2.39, d, *J* = 1.7 Hz/74.5 (CH-1)) and by IR (Infrared) absorption at 3023 (it is the strongest signal) and 2134 cm^−1^.

**Figure 2:**
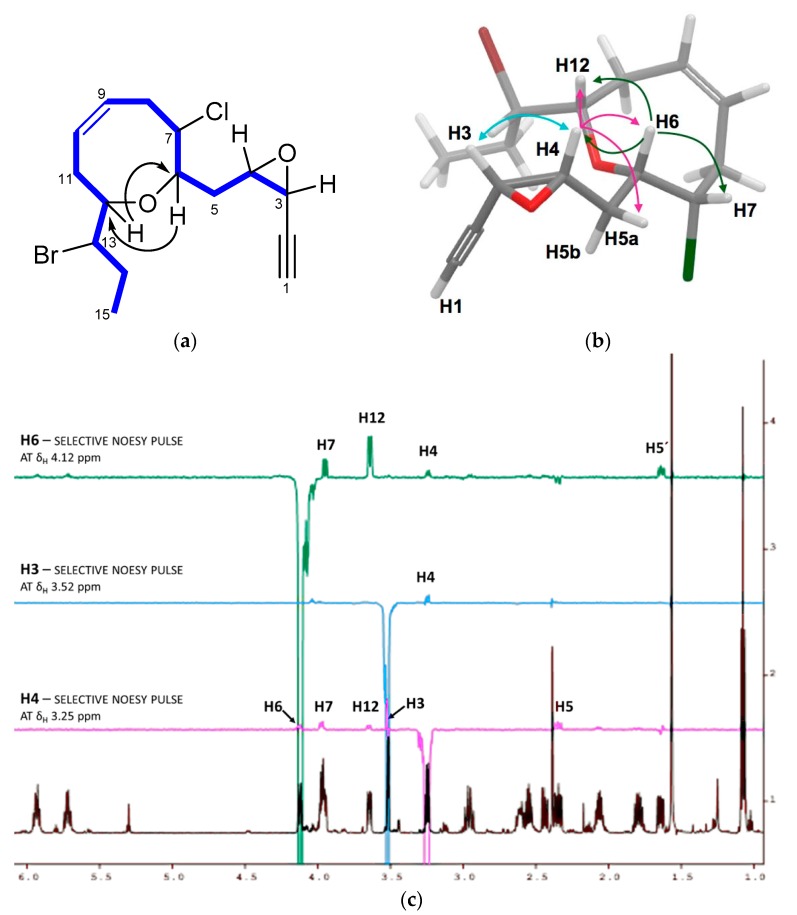
(**a**) C-3–C-15 spin system, correlated by ^1^H-^1^H COSY experiment and selected HMBC correlations of **3**; (**b**) key NOE correlations used to determine the relative configuration; and (**c**) 1D-NOE spectra.

**Figure 3:**
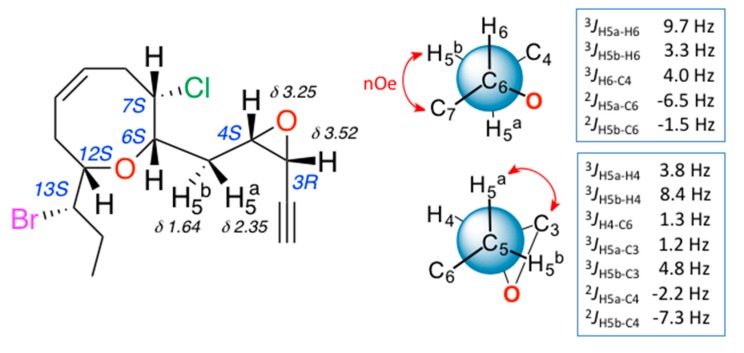
*J*-based configuration analysis for C-4/C-6 fragment of (3*R*,4*S*)-epoxy-pinnatifidenyne (**3**).

The relative configuration of **3** was determined by a combination of NOESY (Nuclear Overhauser Spectroscopy) data and *J*-based configuration approach. 1D-NOE (Nuclear Overhauser Effect) correlations observed between H-6 and H-7/H-12 located all these protons on the same face of the heterocycle (Figure 2), consistent with the orientation previously observed in the pinnatifidenynes. The relationship between the configurations of C-12 and C-13 was established comparing chemical shifts and coupling constants of **3** with those of pinnatifidenynes sharing the same C-15–C-6 fragment [7]. To complete the structural determination, the relative configuration of the epoxide as well as its stereochemical relationship with C-6, the homo- and heteronuclear *J* couplings were measured [15,16]. The ^n^*J*_C,H_ values were accurately obtained using HSQC-HECADE (Heteronuclear Couplings from ASSCI (American Standard Code for Information Interchange)-Domain experiments with E.COSY-type cross peaks) experiment. The value of the coupling constants observed for ^3^*J*_H3-H4_ = 4.0 Hz, confirmed by the observation of a strong dipolar correlation between H-3 and H-4, ensure the *cis* configuration for the epoxide (Figure 2c). Finally, the relative configurations at C-6 and C-4 can be conveniently started from the large coupling constant displayed by the protons H-6 and H-5a (^3^*J*_H6-H5a_ = 9.7 Hz) and the observed value of ^2^*J*_C6-H5b_ = −1.5 Hz that suggested an *anti* relationship for H-6 and H-5a (δ_H_ 2.35) and a *gauche* orientation between H-6 and H-5b (δ_H_ 1.64), confirmed by the dipolar correlation between H-5b and H-7. Similarly, it was established a *threo*-configuration between H-4 and H-5a explained by the observed values of ^3^*J*_H4-H5b_ = 8.4 Hz, ^3^*J*_C3-H5a_ = 1.2 Hz and ^2^*J*_C4-H5a_ = −2.2 Hz. Further support was obtained from additional long-range heteronuclear coupling constants shown in Figure 3.

Compound **4** has the same molecular formula as **3** together with a close structural relationship. Comparison of their ^1^H and ^13^C NMR chemical shifts and the analysis of their 2D NMR data allowed us to establish an identical planar structure for both compounds (see Table 1 and Table 2 and Experimental Section).

**Figure 4:**
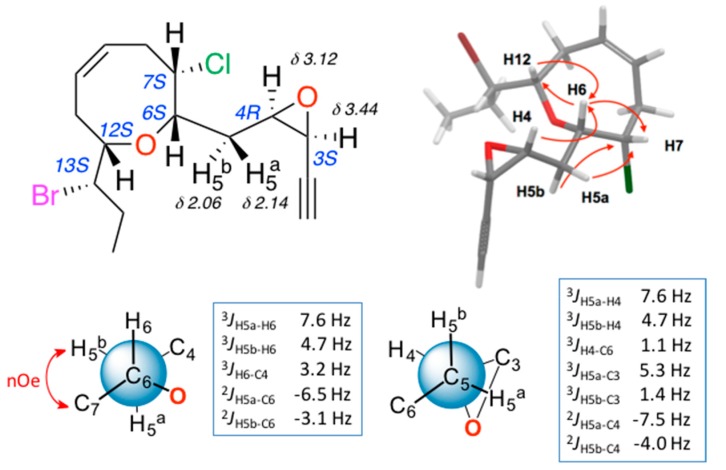
Configuration analysis for (3*S*,4*R*)-epoxy-pinnatifidenyne (**4**).

The stereochemical relationships between the different stereogenic centres, including those of epoxide, were performed using the above-described methods. The results of the NMR configurational analysis are shown in Figure 4. The conclusion was that compounds **3** and **4** share the relative configuration at centers C-6, C-7, C-12 and C-13, as well as the *cis* configuration of the epoxide, while the oxirane rings present an opposite relative configuration.

**Figure 5:**
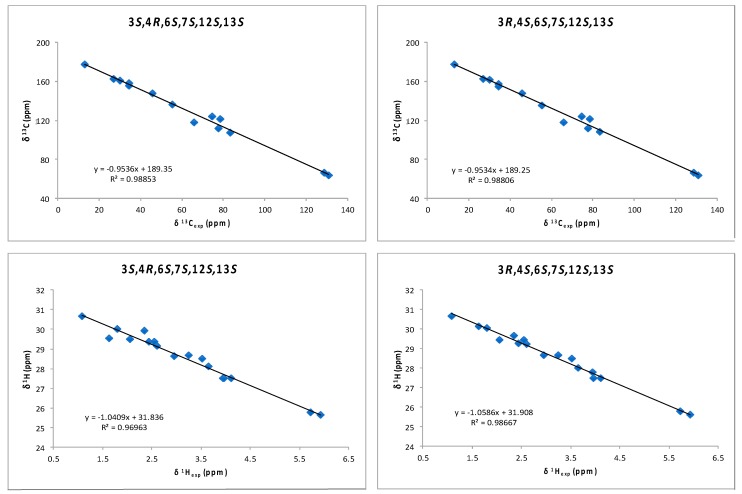
^1^H and ^13^C correlations between calculated isotropic shieldings for the two simulated diastereoisomers of (3,4)-epoxy-pinnatifidenyne and experimentally observed chemical shifts for compound **3**. Fitting parameters are indicated.

**Figure 6:**
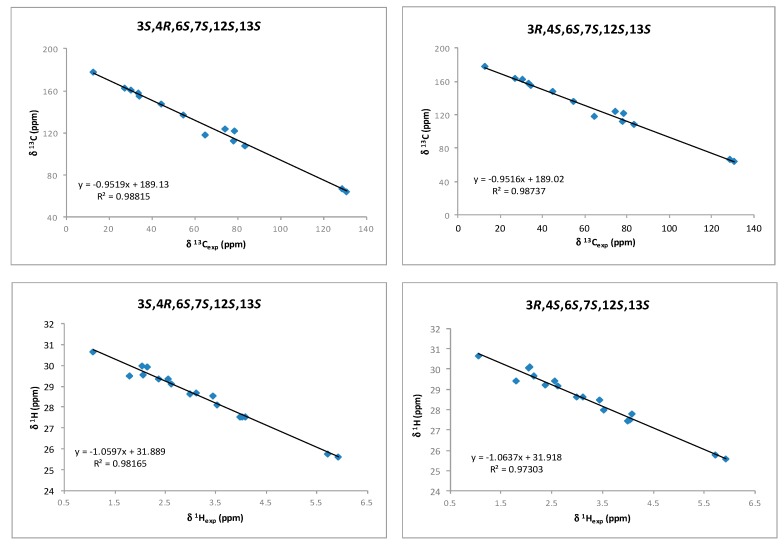
^1^H and ^13^C correlations between calculated isotropic shieldings for the two simulated diastereoisomers of (3,4)-epoxy-pinnatifidenyne and experimentally observed chemical shifts for compound **4**. Fitting parameters are indicated.

Although a *J*-based configurational analysis on **3** and **4** was done to determine its relative configuration, a complementary computational study was undertaken. Chemical shift calculations of carbon atoms attached to halogens are not accurate or difficult to calculate due to spin–orbit contributions. However, Kutateladze et al. [17] have recently reported the use of parametric corrections to calculate ^13^C chemical shifts by DFT using inexpensive computations at the B3LYP/6-31G(d) level for structure optimization and using the ωB97xD/6-31G(d) level for the chemical shift calculations. Using this methodology, we built all possible stereoisomers for compound **3** followed by the corresponding conformational searches, structure optimizations and chemical shift calculations for each calculated conformer. As a result, it turned out that the 3*R*,4*S*,6*S*,7*S*,12*S*,13*S* isomer showed the lowest RMSD (Root Mean Square Deviation) for compounds **3** and **4** (see Supplementary Materials, Table S1). This result was coincident with our NMR-based proposal for the relative configuration of all stereogenic centers within the oxirane ring. However, it was clear that it was unable to solve the uncertainty within the oxirane ring. Taking into account that the previously mentioned methodology uses only ^13^C data, we decided to use chemical shift calculations of ^1^H for a second comparison as it has been shown that the combination of both chemical shifts yields better results [12,13,18,19,20]. Thus, models of the two possible diastereoisomers, 3*S*,4*R*,6*S*,7*S*,12*S*,13*S* (**3a**) and 3*R*,4*S*,6*S*,7*S*,12*S*,13*S* (**3b**), were built and conformational searches on each one, consisting on 5000 steps of a hybrid MCMM (Monte Carlo Multiple Minimum), Low-Mode sampling using the MMFF94 (Merck Molecular Force Field) force field, were completed. Redundant conformers within a 12 kJ/mol energy window of the global minimum found were eliminated using an RMSD cutoff of 1.0 Å. Next, all the resulting structures (seven conformers for **3a** and five conformers for **3b**) were geometrically optimized using DFT calculations [21] at the B3LYP/6-31G** level of theory with the LACVP basis set in gas phase [22]. NMR shielding constants (σ) were calculated according to the calculated relative Boltzmann populations for each conformer. Finally, NMR chemical shifts were obtained scaling the calculated values by linear regression analysis of experimental and computed data. In this analysis, the carbon nucleus with the attached bromine atom and the acetylenic proton were not included due to their high deviations. Correlation coefficients were almost identical for both stereoisomers (0.9885 for **3a** vs. 0.9881 for **3b**) using the ^13^C NMR data but clearly better for isomer **3b** using ^1^H-NMR data (0.9867 vs. 0.9696) (Figure 5 and Supplementary Materials, Table S2). Calculation of the DP4 parameter [23] showed that the 3*R*,4*S*,6*S*,7*S*,12*S*,13*S* diastereoisomer (**3b**) is the most likely solution, with a probability 99.9% using both ^13^C and ^1^H data, therefore supporting our NMR-based proposal. Moreover, the same procedure was applied to fit the data of compound **4** to the calculated values for diasteroisomers **3a** and **3b** (Figure 6 and Supplementary Materials, Table S2). In this case, it turned out that the experimental data of **4** fitted better with calculated data for the 3*S*,4*R*,6*S*,7*S*,12*S*,13*S* diasteroisomer (**3a**), confirming that compounds **3** and **4** are the two-possible *cis* oxirane stereoisomers.

Compound **5** was isolated as an amorphous solid that was determined to have the molecular formula C_15_H_20_BrClO_2_ by HR-ESI-MS analysis. The presence in **5** of a terminal acetylene group conjugated with a double bond was evident from UV (Ultra Violet) [λ_max_ (log *ε*) 225 (3.59) nm] and IR [ν_max_ 3289 and 2300 cm^−1^] spectra. The ^1^H NMR experiment showed the characteristic shift signals at δ_H_ 3.17 (1H, br.s), 5.63 (1H, br.d, *J* = 10.8 Hz) and 6.05 (1H, ddd, *J* = 7.6, 7.7, 10.8 Hz) of the *Z*-enyne unit clearly related to *Z*-pinnatifidenyne (**1**). An extensive assessment of 1D and 2D NMR data (Table 2 and Table 3) compared to reported data for **1** [7] indicated that compound **5** differs to **1** containing an extra disubstituted epoxide situated at C-9–C-10. The relative configuration of **5** was determined by observation of dipolar correlations. Accordingly, the observed NOE enhancements from H-10 to H-9 and H-12, and from H-6 to H-7 and H-12 located all these protons on the same face of the molecule (Supplementary Materials, Figure S23). The above data and the coupling constant for ^3^*J*_H9-H10_ = 4.0 Hz provide evidence of the *cis*-orientation. This structure corresponds to a previously reported synthetic epoxide obtained by epoxidation of **1** and published by our group in 1982 [7]. However, the data published at that time and those obtained for our natural product did not correlate well. Therefore, to confirm the structure of **5** as (9*R*,10*S*)-epoxy-*Z*-pinnatifidenyne, we decided to repeat the selective epoxidation of **1**. A product identical to **5** in all respects was obtained (see Experimental part).

Pinnatifidehyde **6** is a yellow amorphous solid that shows the molecular formula C_12_H_18_BrClO_2_, evidenced by the presence of three pseudomolecular [M + Na]^+^ ions in the HR-ESI-MS spectrum at *m*/*z* 331.0086, 333.0056 and 335.0033 (ratio 74:100:36, calcd. 331.0076, 333.0047, 335.0026). 

^1^H and ^13^C NMR spectra (Table 2 and Table 3) were reminiscent of the corresponding partial spectral signals of pinnatifidenynes **1**–**2** or compounds **3**–**4**. All those compounds share identical oxacyclic and bromopropyl terminal chain (C-6→C-15 moiety), whereas the only notable differences were fixed going towards C-4 were the structure appears truncated. Thus, the deshielded signals of a methylene at δ_H_ 3.10 (br.dd, *J* = 8.0, 18.7 Hz) and 2.71 (dd, *J* = 4.0, 18.7 Hz), as well as the methine at δ_H_ 4.46 (ddd, *J* = 2.9, 4.0, 8.0 Hz), equivalent respectively to H_2_-5 and H-6 in pinnatifidenynes, showed correlations in the HMBC with a carbonyl signal at δ_C_ 200.2 corresponding to one aldehyde group. The structure of compound **6** possesses a truncated C_12_ carbon skeleton that may be derived from compounds **1** or **2** based on a probable oxidative process. Finally, analysis of the ROESY experiment confirmed the relative configuration of all stereogenic centers presented in the molecule as equivalent to those observed in the pinnatifidenynes (**1**, **2**). Pinnatifidehyde **6** has been a synthetic intermediate target in the total synthesis of compounds **1** and **2** [10,24]. The spectroscopic data of synthesized intermediate by the Snyder group [10] was in agreement with the spectroscopic data of the natural product **6**.

**Figure 7:**
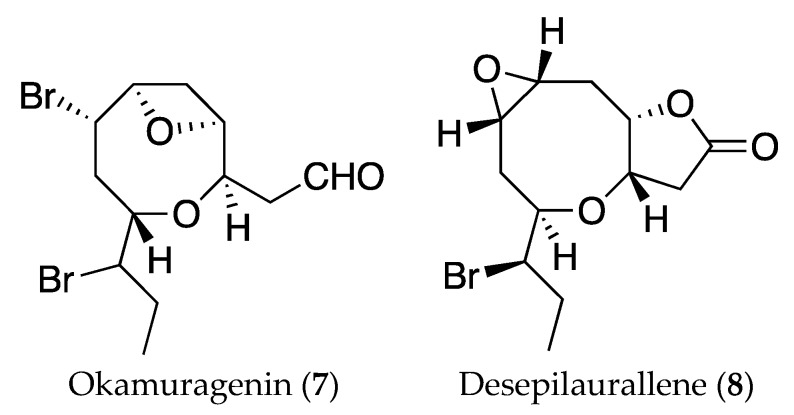
Structures of C_12_ metabolites isolated from *Laurencia okamurai*, okamuragenin (**7**) and desepilaurallene (**8**).

As far as we know, pinnatifidehyde (**6**) is the third example of C_12_ acetogenins; the other two, okamuragenin (**7**) and desepilaurallene (**8**) (Figure 7), have been isolated from *Laurencia okamurai* [25,26]. It has to be noted that the new compounds with the ethynyl oxirane unit (**3** and **4**) could be considered biogenetic precursors of pinnatifidehyde.

The antiproliferative activity against six representative human solid tumor cell lines was evaluated for compounds **3**–**6** [27]. The results showed that compound **5** was the most potent compound of the series, with a modest activity against four of the cell lines tested (GI_50_ 13–48 μM). Compound **4** was active against two of the cell lines (GI_50_ 33–45 μM), whilst the remaining compounds were inactive (GI_50_ > 50 μM) (see Supplementary Materials, Table S15).

## 3. Materials and Methods 

### 3.1. General Experimental Procedures

Optical rotations were measured at room temperature in CHCl_3_ on a PelkinElmer-241 polarimeter (Waltham, MA, USA) by using a sodium lamp. IR spectra were recorded on a Bruker IFS55 spectrophotometer (Ettlingen, Germany) using methanolic solutions over NaCl disk. NMR spectra were recorded on a Bruker Avance 600 instrument (Karlsruhe, Germany) equipped with a 5-mm TCI (Triple Resonance CryoProbe) inverse detection cryo-probe. ^1^H and ^13^C NMR chemical shifts were referenced either to the CDCl_3_ or C_6_D_6_ solvent peaks at 300 K (CDCl_3_: δ_H_ 7.26, δ_C_ 77.0). COSY, HSQC, HMBC and ROESY experiments were performed using standard pulse sequences. ^3^*J*_H,H_ values were measured from 1D ^1^H NMR. The HSQC-HECADE pulse sequence was used to measure long-range heteronuclear coupling constants. All experiments were performed in the phase-sensitive mode (States-TPPI (Time-Proportional Phase-Incrementation frequency discrimination) or echo-antiecho for quadrature detection in F1) and used gradient coherence selection. The HSQC-HECADE experiment was recorded using DIPSI (Decoupling in the Presence of Scalar Interactions) during the 40 ms of the isotropic mixing period using a bandwidth of 10 kHz, and a *J*-scale factor of 1 was used. Prior to Fourier transformation, zero filling was performed to expand the data to at least double the number of acquired data points. HR-ESI-MS data were obtained on a LCT Premier XE Micromass spectrometer (Waters, Milford, CT, USA). HPLC (High performance liquid chromatography) separations were carried out with a LKB 2248 system (Bromma, Sweden) equipped with a photodiode array detector. TLC (Thin layer chromatography) (Merck, Darmstadt, Germany) was visualized by spraying with phosphomolybdic acid reagent (10% in EtOH) and heating. 

### 3.2. Computational Methods

Conformational searches were undertaken using the Macromodel software (version 8.5, Schrödinger Inc., San Diego, CA, USA) and the MMFF94 force field. Solvation effects of CHCl_3_ were simulated using the generalized Born/surface area (GBSA) solvation model. Extended non-bonded cutoff distances (a van der Waals cutoff of 8.0 Å and an electrostatic cutoff of 20.0 Å) were used. Local minima within 10 kJ of the global minimum were saved and analysis of the results was undertaken using Maestro software. Quantum mechanical calculations were carried out with the Jaguar package (Jaguar; Schrödinger LLC, New York, NY, USA). Single-point energy calculations were performed at the DFT theoretical level in the gas phase. The B3LYP hybrid functional with the LACVP ** basis set was used. Chemical shifts were calculated using the gauge-including atomic orbital (GIAO) method. Chemical shifts were calculated from their shielding constants that were first averaged according to their relative Boltzmann populations using a Schrödinger Inc. python script. Proton chemical shifts for each methyl group were averaged due to their conformational freedom.

### 3.3. Biological Material 

Specimens of *Laurencia viridis* were collected by hand in the intertidal zone at Callao Salvaje, Tenerife, Canary Islands, Spain (28°07′12′′ N, 16°46′45′′ W) in April 2013. A voucher specimen was deposited at the Department of Biología Vegetal, Botánica, University of La Laguna, Tenerife (TFC Phyc 7180, Herbarium Code of University of La Laguna).

### 3.4. Extraction and Isolation

Fresh specimens were extracted at room temperature using CHCl_3_: CH_3_OH (1:1, *v*/*v*). The resulting extract (77.5 g) was separated in Silicagel 0.2–0.5 mm (Sigma-Aldrich, St. Louis, MO, USA) using a gradient of *n*-hexane:ethyl acetate. The fraction corresponding at 10% of *n*-hexane was chromatographed in Sephadex LH-20 (Sigma, St. Louis, MO, USA) (*n*-hexane:CHCl_3_:CH_3_OH, 2:1:1). The first fraction was treated in Sephadex LH-20 in the same conditions, yielding eight fractions. Selected fractions that exhibits similar TLC profiles were rechromatographed on normal phase open column (Silicagel, 0.2–0.5 mm), using a gradient of *n*-hexane:acetone from 49:1 to 1:1, yielding compound **6** (3.2 mg) in the second fraction. The compounds **3**–**5** were isolated and purified from the first fraction by HPLC using µ-Porasil™ HPLC column (Waters, Milford, CT, USA), 10 µm, 19 × 150 mm, and *n*-hexane:ethyl acetate (49:1) as mobile phase (Supplementary Materials, Scheme S1).

(3*R*,4*S*)-Epoxy-pinnatifidenyne (**3**): yellow, amorphous substance; ${\left[\alpha \right]}_{D}^{25}$ −17 (*c* 0.09, CHCl_3_); IR ν_max_ (CHCl_3_) 3023, 2788, 2379, 2134, 1748, 1698, 1647, 1571, 1500, 1401, 1311, 1189, 1098 cm^−1^; ^1^H and ^13^C NMR data (CDCl_3_), see Table 1 and Table 2; HR-ESI-MS *m*/*z* 369.0227, 371.0216 and 373.0201 [M + Na]^+^ (76:100:24) (calcd. for C_15_H_20_O_2_^35^Cl^79^BrNa, 369.0233; C_15_H_20_O_2_^37^Cl^79^BrNa, 371.0203; C_15_H_20_O_2_^35^Cl^81^BrNa, 371.0212; C_15_H_20_O_2_^37^Cl^81^BrNa, 373.0183).

(3*S*,4*R*)-Epoxy-pinnatifidenyne (**4**): yellow, amorphous solid; ${\left[\alpha \right]}_{D}^{25}$ +30 (*c* 0.1, CHCl_3_); IR ν_max_ (CHCl_3_) 3289, 2935, 2117, 1743, 1454, 1384, 1103 cm^−1^; ^1^H and ^13^C NMR data (CDCl_3_), see Table 1 and Table 2; HR-ESI-MS *m*/*z* 369.0222, 371.0204 and 373.0195 [M + Na]^+^ (79:100:31) (calcd. for C_15_H_20_O_2_^35^Cl^79^BrNa, 369.0233; C_15_H_20_O_2_^37^Cl^79^BrNa, 371.0203; C_15_H_20_O_2_^37^Cl^81^BrNa, 371.0212; C_15_H_20_O_2_^37^Cl^81^BrNa, 373.0183). 

(9*R*,10*S*)-Epoxy-*Z*-pinnatifidenyne (**5**): yellow, amorphous solid; ${\left[\alpha \right]}_{D}^{25}$ +8 (*c* 0.24, CHCl_3_); IR ν_max_ (CHCl_3_) 3289, 2935, 2300, 2015, 1743, 1454, 1379, 1103 cm^−1^; ^1^H and ^13^C NMR data (CDCl_3_), see Table 1 and Table 2; HR-ESI-MS *m*/*z* 369.0219, 371.0214 and 373.0197 [M + Na]^+^ (84:100:25) (calcd. for C_15_H_20_O_2_^35^Cl^79^BrNa, 369.0233; C_15_H_20_O_2_^37^Cl^79^BrNa, 371.0203; C_15_H_20_O_2_^35^Cl^81^BrNa, 371.0212; C_15_H_20_O_2_^37^Cl^81^BrNa, 373.0183).

Pinnatifidehyde (**6**): yellow, amorphous substance; ${\left[\alpha \right]}_{D}^{25}$ +33 (*c* 0.3, CHCl_3_); IR ν_max_ (CHCl_3_) 3021, 2971, 2881, 2181, 2157, 2010, 1965, 1751, 1700, 1645, 1610, 1500, 1312, 1193, 1099 cm^−1^; ^1^H and ^13^C NMR data (CDCl_3_), see Table 1 and Table 2; HR-ESI-MS *m*/*z* 331.0086, 333.0056 and 335.0033 [M + Na]^+^ (74:100:36) (calcd. for C_12_H_18_O_2_^35^Cl^79^BrNa, 331.0076; C_12_H_18_O_2_^37^Cl^79^BrNa, 333.0047; C_12_H_18_O_2_^35^Cl^81^BrNa, 333.0047; C_12_H_18_O_2_^37^Cl^81^BrNa, 335.0026).

### 3.5. Chemical Conversion of Z-Pinnatifidenyne (***1***) to (9R,10S)-Epoxy-Z-Pinnatifidenyne (***5***)

m-Chloroperbenzoic acid (26 mg) in dry benzene (1.2 mL) was added dropwise to a solution of *Z*-pinnatifidenyne (23 mg) in benzene (0.84 mL) with stirring [7]. The reaction was led overnight and in the final step calcium hydroxide was added to remove the excess of peracid. The solution was then filtered and evaporated. The residue obtained was applied in to a silica gel (230–400 mesh, 60 Å) column eluted whit ethyl acetate:*n*-hexane gradient (2:8 to 3:7) yielding (9*R*,10*S*)-epoxy-*Z*-pinnatifidenyne (2.4 mg).

### 3.6. Antiproliferative Activity

Human solid tumor cell lines A549, HBL-100, HeLa, SW1573, T-47D and WiDr were a kind gift from Prof. G. J. Peters (VU Medical Center, Amsterdam, The Netherlands). The cell lines were cultured in RPMI 1640 medium (Flow Laboratories, Irvine, UK), supplemented with 5% fetal calf serum (FCS, Gibco, Grand Island, NY, USA), 2 mM l-glutamine (Merck, Darmstadt, Germany), 100 U/mL of penicillin G and 0.1 mg/mL of streptomycin (Sigma, St. Louis, MO, USA) at 37 °C in a 95% humidified with 5% CO_2_ atmosphere. The in vitro antiproliferative activity was evaluated using the sulforhodamine B (SRB, Sigma, St. Louis, MO, USA) assay with slight modifications [27]. Briefly, pure compounds were initially dissolved in DMSO (Sigma, St. Louis, MO, USA) at 400 times the desired final maximum test concentration. Cells were inoculated onto 96-well plates in a volume of 100 μL per well at densities of 2500 (A549, HBL-100, HeLa and SW1573) or 5 000 (T-47D and WiDr) cells per well, based on their doubling times. Control cells were exposed to an equivalent concentration of DMSO (0.25% *v*/*v*, negative control). Each agent was tested in triplicate at different dilutions in the range of 0.5–50 µM. The drug treatment was started on day 1 after plating. Drug incubation times comprised 48 h, after which time cells were precipitated with 25 µL ice-cold 50% (*w*/*v*) trichloroacetic acid (Merck, Darmstadt, Germany) and fixed for 60 min at 4 °C. Then the SRB assay was performed. The optical density (OD) of each well was measured at 530 nm, using BioTek’s PowerWave XS microplate reader. Values were corrected for background OD from wells containing only medium. The percentage growth (PG) was calculated in relation to untreated control cells (C) at each of the drug concentration levels based on the difference in OD at the start (T_0_) and end of drug exposure (T), according to NCI formulas Therefore, if T is greater than or equal to T_0_, the calculation is 100 × [(T − T_0_)/(C − T_0_)]. If T is less than T_0_, denoting cell killing, the calculation is 100 × [(T − T_0_)/(T_0_)]. The effect is defined as percentage of growth, where 50% growth inhibition (GI_50_) represents the concentration at which PG is +50. With these calculations, a PG value of 0 represents no difference from the start of drug exposure, while negative PG values denote net cell death.

## Figures and Tables

**Table 1:** ^1^H NMR data for compounds **3** and **4** (600 MHz, CDCl_3_).

Position	(3*R*,4*S*)-Epoxy-Pinnatifidenyne (3)	(3*S*,4*R*)-Epoxy-Pinnatifidenyne (4)
	δ_H_ (*J* in Hz)	δ_H_ (*J* in Hz)
**1**	2.39, d (1.7)	2.38, d (1.7)
**3**	3.52, dd (1.7, 4.0)	3.44, dd (1.7, 4.0)
**4**	3.25, ddd (3.8, 4.0, 8.4)	3.12, ddd (4.0, 4.7, 7.6)
**5**	1.64, ddd (3.3, 8.4, 14.6) 2.35, ddd (3.8, 9.7, 14.6)	2.06, ddd (4.7, 7.0, 14.5) 2.14, ddd (7.6, 7.6, 14.5)
**6**	4.12, ddd (2.5, 3.3, 9.7)	4.03, ddd (2.6, 4.7, 7.6)
**7**	3.95, ddd (2.5, 5.0, 11.6)	4.08, ddd (2.6, 5.1,11.7)
**8**	2,55, ddd (5.0, 6.6, 11.8) 2.96, ddd (1.7, 11.6, 11.8)	2.56, ddd (5.8, 6.1, 12.5) 2.99, dddd (1.8, 11.6, 12.5)
**9**	5.72, dddd (1.7, 6.6, 10.1)	5.72, ddd (1.8, 6.1, 10.3)
**10**	5.93, br.dd (8.4, 10.1)	5.93, br.dd (8.5, 10.3)
**11**	2.44, ddd (1.3, 8.4, 14.0) 2.61, br.dd (3.7, 14.0)	2.37, ddd (1.0, 8.5, 13.9) 2.62, br.dd (3.8, 13.9)
**12**	3.65, ddd (1.3, 3.7, 10.0)	3.53, ddd (1.0, 3.8, 10.1)
**13**	3.97, ddd (3.0, 3.3, 10.0)	3.97, ddd (2.8, 3.5, 10.1)
**14**	1.79, ddq (3.3, 7.2, 14.5) 2.06, ddq (3.0, 7.2, 14.5)	1.80, ddq (2.8, 7.2, 14.5) 2.04, ddq (3.5, 7.2, 14.5)
**15**	1.08, t (7.2)	1.08, t (7.2)

**Table 2:** ^13^C NMR data for compounds **3**–**6** (150 MHz, CDCl_3_).

Position		3	4	5	6
	Multi.	δ_C_	δ_C_	δ_C_	δ_C_
**1**	CH	74.5	74.3	83.1	
**2**	C	78.6	78.6	80.5	
**3**	CH	45.6	44.7	111.7	
**4**	CH	55.3	54.8	140.2	200.2
**5**	CH_2_	34.3	33.7	35.1	49.1
**6**	CH	77.5	78.1	81.5	74.2
**7**	CH	65.8	64.8	60.3	65.4
**8**	CH_2_	34.5	34.4	34.5	34.4
**9**	CH	128.9	128.9	52.6	128.7
**10**	CH	131.0	131.0	52.9	131.3
**11**	CH_2_	30.0	30.4	32.1	31.2
**12**	CH	83.1	83.5	81.8	83.3
**13**	CH	61.1	61.2	59.9	61.8
**14**	CH_2_	27.1	27.3	26.8	27.7
**15**	CH_3_	12.8	12.9	12.9	12.8

**Table 3:** ^1^H NMR data for compounds **5** and **6** (600 MHz, CDCl_3_).

Position	(9*R*,10*S*)-Epoxy-*Z*-Pinnatifidenyne (5)	Pinnatifidehyde (6)
	δ_H_ (*J* in Hz)	δ_H_ (*J* in Hz)
**1**	3.17, br.s	
**3**	5.63, br.d (10.8)	
**4**	6.05, ddd (7.6, 7.7, 10.8)	9.80, br.s
**5**	2.58, dd (5.5, 7.7, 14.2) 2.87, ddd (7.6, 7.6, 14.2)	2.71, dd (4.0, 18.7) 3.10, br.dd (8.0, 18.7)
**6**	3.79, ddd (2.3, 5.5, 7.6)	4.46, ddd (2.9, 4.0, 8.0)
**7**	4.11, ddd (2.3, 4.8, 11.7)	4.00, ddd (2.9, 5.1, 11.8)
**8**	1.94, ddd (11.6, 11.7, 13.2) 2.67, ddd (3.5, 4.8, 13.2)	2.53, ddd (2.2, 5.1, 12.0) 2.96, ddd (5.1, 6.6, 12.0)
**9**	2.88, ddd (3.5, 4.0, 11.6)	5.72, ddd (2.2, 6.6, 10.3)
**10**	3.05, ddd (4.0, 4.6, 9.4)	5.96, br.dd, (8.3, 10.3)
**11**	1.65, ddd (9.4, 10.9, 14.0) 2.58, ddd (3.3, 4.6, 14.0)	2.31, ddd, (1.4, 8.3, 14.0) 2.64, br.dd, (3.9, 14.0)
**12**	3.66, dd (3.3; 10.9)	3.67, ddd (1.4, 3.9, 10.3)
**13**	4.00, ddd (3.1, 3.1, 10.9)	3.84, ddd (2.8, 2.9, 10.3)
**14**	1.73, ddq (3.1, 7.3, 14.5) 2.02, ddq (3.1, 7.3, 14.5)	1.74, ddq (2.9, 7.3, 14.6) 1.90, ddq (2.8, 7.3, 14.6)
**15**	1.08, t (7.3)	1.04, t (7.3)

## Supplementary Materials

The following are available online at www.mdpi.com/1660-3397/16/1/5/s1. Scheme S1: Isolation procedure for new acetogenins; Figures S1–S31: NMR and HRESIMS spectra of isolated compounds; Figures S32 and S33: Correlations plots for ^13^C and ^1^H calculated vs. ^13^C and ^1^H experimental data for compounds **3** and **4**; Figure S34: DP4 app for (3*R*,4*S*)-epoxy-pinnatifidenyne **3**; Table S1: Calculated vs. experimental RMDS for compounds **3** and **4**; Table S2: Scaled ^13^C and ^1^H chemical shifts for the two studied diasteroisomers on B3LYP-D3 in gas phase; Tables S3–S14: Computational calculation results; Table S15: Antiproliferative activity against human cancer cell lines.
