# Supplementary material for: Pinnatifidenyne-Derived Ethynyl Oxirane Acetogenins from Laurencia viridis
Source: Mar Drugs. 2017 Dec 29;16(1):5. doi: 10.3390/md16010005 (PMC5793053; doi:10.3390/md16010005)
Supplement: Supplementary file 1 [file marinedrugs-16-00005-s001.pdf]

Supporting Information

Pinnatifidenyne-derived ethynyl oxirane acetogenins from *Laurencia viridis*

Adrián Morales-Amador [1,2], Caterina R. de Vera [1,2], Olivia Márquez-Fernández[3], Antonio Hernández Daranas [1,4], José M. Padrón [1], José J. Fernández [1,2,*], María L. Souto [1,2,*] and Manuel Norte [1,2,*]

[1] Instituto Universitario de Bio-Orgánica Antonio González (IUBO AG), Centro de Investigaciones Biomédicas de Canarias (CIBICAN), Universidad de La Laguna (ULL), Avenida Astrofísico Francisco Sánchez 2, 38206, Tenerife, Spain; E-Mails: adrian_tf@hotmail.com (A.M.-A.); caterina.rdv@gmail.com (C.R.V.); adaranas@ull.es (A.H.D.); jmpadron@ull.es (J.M.P.); jjfercas@ull.es (J.J.F.); msouto@ull.es (M.L.S.); mnorte@ull.es (M.N.)

[2] Departamento de Química Orgánica, Universidad de La Laguna (ULL), Avenida Astrofísico Francisco Sánchez s/n, 38206, Tenerife, Spain

[3] Facultad de Ciencias Biológicas-Universidad Veracruzana, Circuito Gonzalo Aguirre Beltrán s/n, Zona Universitaria C.P. 91090, Xalapa, Veracruz, México; E-Mail: mafo68@yahoo.com.mx (O.M.-F.)

[4] Instituto de Productos Naturales y Agrobiología (IPNA), CSIC, Avenida Astrofísico Francisco Sánchez 2, 38206, Tenerife, Spain

Table of contents

	Page
**Scheme S1.** Isolation procedure for new acetogenins.	4
**Figure S1.** ^{1}H-NMR spectrum (600 MHz, $CDCl_3$) for (3*R*,4*S*)-epoxy-pinnatifidenyne **3**.	5
**Figure S2.** ^{13}C-NMR spectrum (150 MHz, $CDCl_3$) for (3*R*,4*S*)-epoxy-pinnatifidenyne **3**.	6
**Figure S3.** COSY ^{1}H-^{1}H spectrum (600 MHz, $CDCl_3$) for (3*R*,4*S*)-epoxy-pinnatifidenyne **3**.	7
**Figure S4.** HSQCed spectrum (600 MHz, $CDCl_3$) for (3*R*,4*S*)-epoxy-pinnatifidenyne **3**.	8
**Figure S5.** HMBC spectrum (600 MHz, $CDCl_3$) for (3*R*,4*S*)-epoxy-pinnatifidenyne **3**.	9
**Figure S6.** ROESY spectrum (600 MHz, $CDCl_3$) for (3*R*,4*S*)-epoxy-pinnatifidenyne **3**.	10
**Figure S7.** HSQC-HECADE spectrum (600 MHz, $CDCl_3$) for (3*R*,4*S*)-epoxy-pinnatifidenyne **3**.	11
**Figure S8.** 1D NOESY spectra (600 MHz, $CDCl_3$) for (3*R*,4*S*)-epoxy-pinnatifidenyne **3**.	12
**Figure S9.** HRESIMS spectrum for (3*R*,4*S*)-epoxy-pinnatifidenyne **3**.	13
**Figure S10.** ^{1}H-NMR spectrum (600 MHz, $CDCl_3$) for (3*S*,4*R*)-epoxy-pinnatifidenyne **4**.	14
**Figure S11.** ^{13}C-NMR spectrum (150 MHz, $CDCl_3$) for (3*S*,4*R*)-epoxy-pinnatifidenyne **4**.	15
**Figure S12.** HSQCed spectrum (600 MHz, $CDCl_3$) for (3*S*,4*R*)-epoxy-pinnatifidenyne **4**.	16
**Figure S13.** COSY ^{1}H-^{1}H spectrum (600 MHz, $CDCl_3$) for (3*S*,4*R*)-epoxy-pinnatifidenyne **4**.	17
**Figure S14.** HMBC spectrum (600 MHz, $CDCl_3$) for (3*S*,4*R*)-epoxy-pinnatifidenyne **4**.	18
**Figure S15.** ROESY spectrum (600 MHz, $CDCl_3$) for (3*S*,4*R*)-epoxy-pinnatifidenyne **4**.	19
**Figure S16.** 1D NOESY spectra (600 MHz, $CDCl_3$) for (3*S*,4*R*)-epoxy-pinnatifidenyne **4**.	20
**Figure S17.** HRESIMS spectrum for (3*S*,4*R*)-epoxy-pinnatifidenyne **4**.	21
**Figure S18.** ^{1}H-NMR spectrum (600 MHz, $CDCl_3$) for (9*R*,10*S*)-epoxy-*Z*-pinnatifidenyne **5**.	22
**Figure S19.** HSQCed spectrum (600 MHz, $CDCl_3$) for (9*R*,10*S*)-epoxy-*Z*-pinnatifidenyne **5**.	23
**Figure S20.** COSY ^{1}H-^{1}H spectrum (600 MHz, $CDCl_3$) for (9*R*,10*S*)-epoxy-*Z*-pinnatifidenyne **5**.	24
**Figure S21.** HMBC spectrum (600 MHz, $CDCl_3$) for (9*R*,10*S*)-epoxy-*Z*-pinnatifidenyne **5**.	25
**Figure S22.** ROESY spectrum (600 MHz, $CDCl_3$) for (9*R*,10*S*)-epoxy-*Z*-pinnatifidenyne **5**.	26
**Figure S23.** 1D NOESY spectra (600 MHz, $CDCl_3$) for (9*R*,10*S*)-epoxy-*Z*-pinnatifidenyne **5**.	27
**Figure S24.** HRESIMS spectrum for (9*R*,10*S*)-epoxy-*Z*-pinnatifidenyne **5**.	28
**Figure S25.** ^{1}H-NMR spectrum (600 MHz, $CDCl_3$) for pinnatifidehyde **6**.	29
**Figure S26.** ^{13}C-NMR spectrum (125 MHz, $CDCl_3$) for pinnatifidehyde **6**.	30
**Figure S27.** COSY ^{1}H-^{1}H spectrum (600 MHz, $CDCl_3$) for pinnatifidehyde **6**.	31
**Figure S28.** HSQC spectrum (600 MHz, $CDCl_3$) for pinnatifidehyde **6**.	32
**Figure S29.** HMBC spectrum (600 MHz, $CDCl_3$) for pinnatifidehyde **6**.	33
**Figure S30.** ROESY spectrum (600 MHz, $CDCl_3$) for pinnatifidehyde **6**.	34
**Figure S31.** HRESIMS spectrum for pinnatifidehyde **6**.	35
**Table S1.** Calculated *vs* experimental RMSD for compounds **3** and **4**.	36
**Table S2.** Scaled ^{13}C and ^{1}H chemical shifts for the two studied diastereoisomers on B3LYP-D3 in gas phase.	37
**Figure S32.** Correlations plots for ^{13}C and ^{1}H calculated *vs* ^{13}C and ^{1}H experimental data for (3*R*,4*S*)-epoxy-pinnatifidenyne **3**.	38
**Figure S33.** Correlations plots for ^{13}C and ^{1}H calculated *vs* ^{13}C and ^{1}H experimental data for (3*S*,4*R*)-epoxy-pinnatifidenyne **4**.	39
**Table S3.** Conformer 1 of 12 (RS_1).	40
**Table S4.** Conformer 2 of 12 (RS_2).	41
**Table S5.** Conformer 3 of 12 (RS_7).	42
**Table S6.** Conformer 4 of 12 (RS_11).	43

**Table S7.** Conformer 5 of 12 (RS_12).	44
**Table S8.** Conformer 6 of 12 (RS_12).	45
**Table S9.** Conformer 7 of 12 (SR_1).	46
**Table S10.** Conformer 8 of 12 (SR_10) .	47
**Table S11.** Conformer 9 of 12 (SR_9).	48
**Table S12.** Conformer 10 of 12 (RS_2).	49
**Table S13.** Conformer 11 of 12 (SR_6).	50
**Table S14.** Conformer 12 of 12 (SR_12).	51
**Figure S34.** DP4 app for (3*R*,4*S*)-epoxy-pinnatifidenyne **3**.	52
**Table S15.** Antiproliferative activity against human cancer cell lines.	53

Scheme S1. Isolation procedure for new acetogenins.

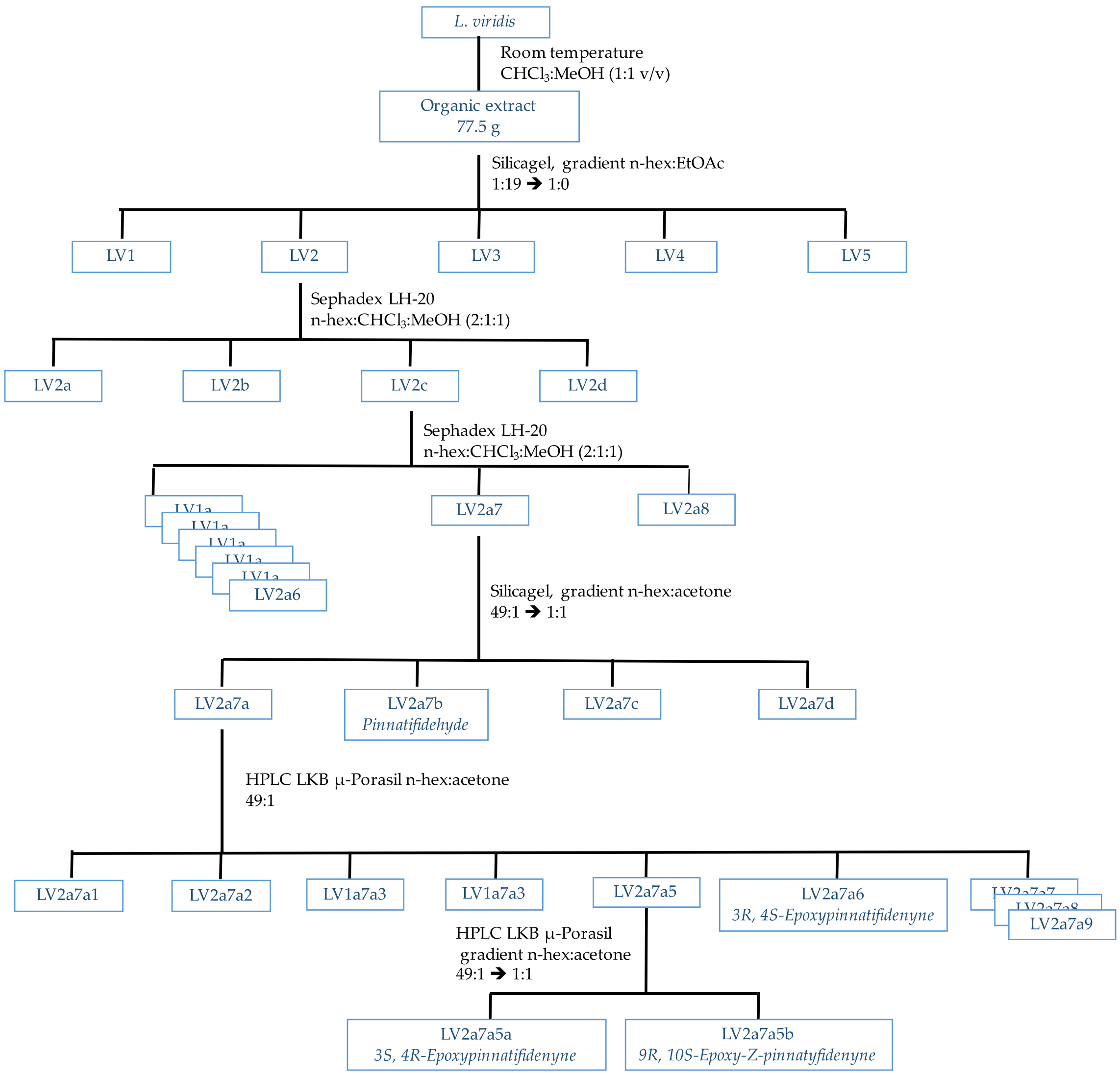

Figure S1. ^{1}H-NMR spectrum (600 MHz, $CDCl_3$) for (3*R*,4*S*)-epoxy-pinnatifidenyne **3**.

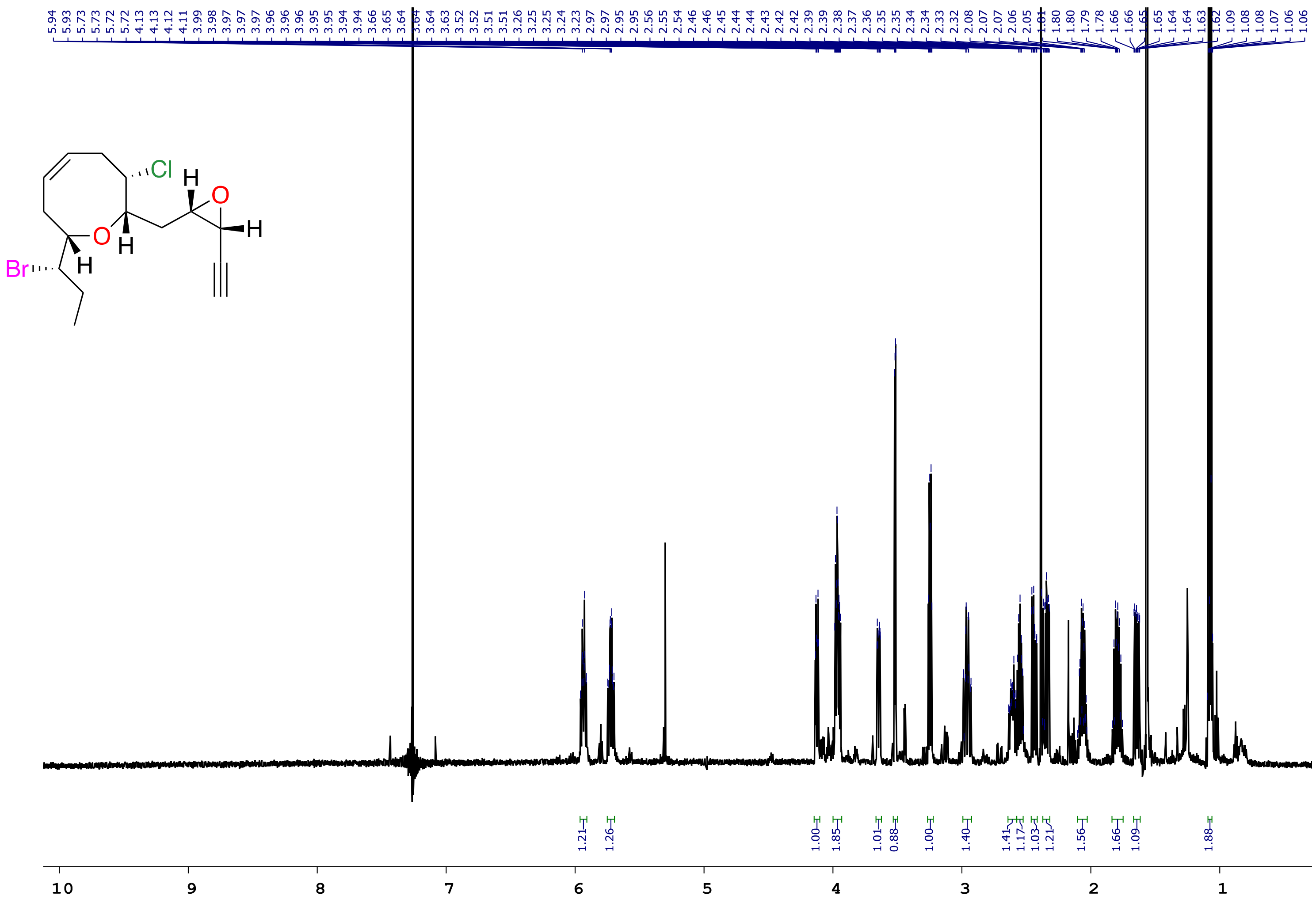

Figure S2. ^{13}C-NMR spectrum (150 MHz, $CDCl_3$) for (3*R*,4*S*)-epoxy-pinnatifidenyne **3**.

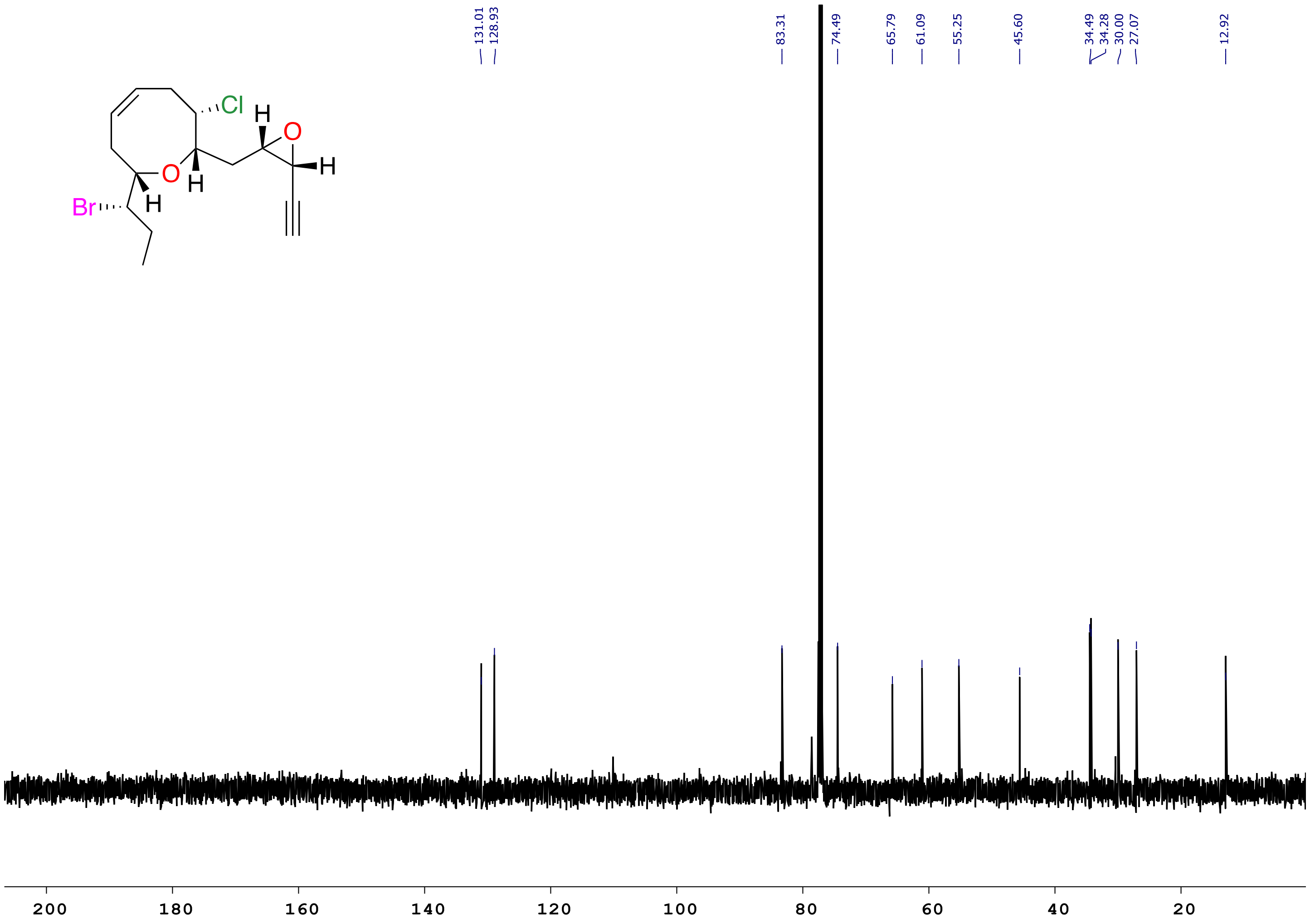

Figure S3. COSY ^{1}H-^{1}H spectrum (600 MHz, $CDCl_3$) for (3*R*,4*S*)-epoxy-pinnatifidenyne **3**.

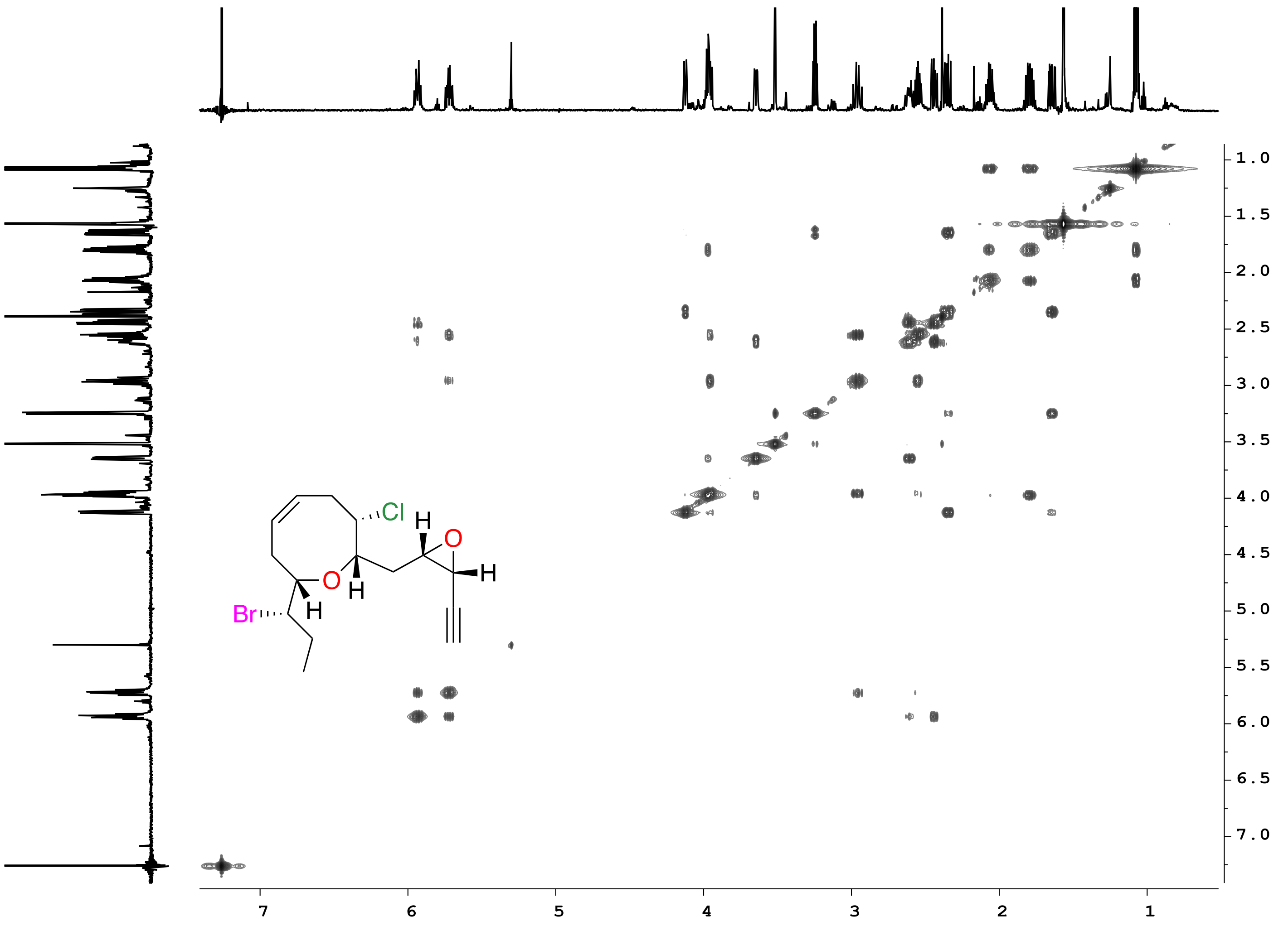

Figure S4. HSQCed spectrum (600 MHz, $CDCl_3$) for (3*R*,4*S*)-epoxy-pinnatifidenyne **3**.

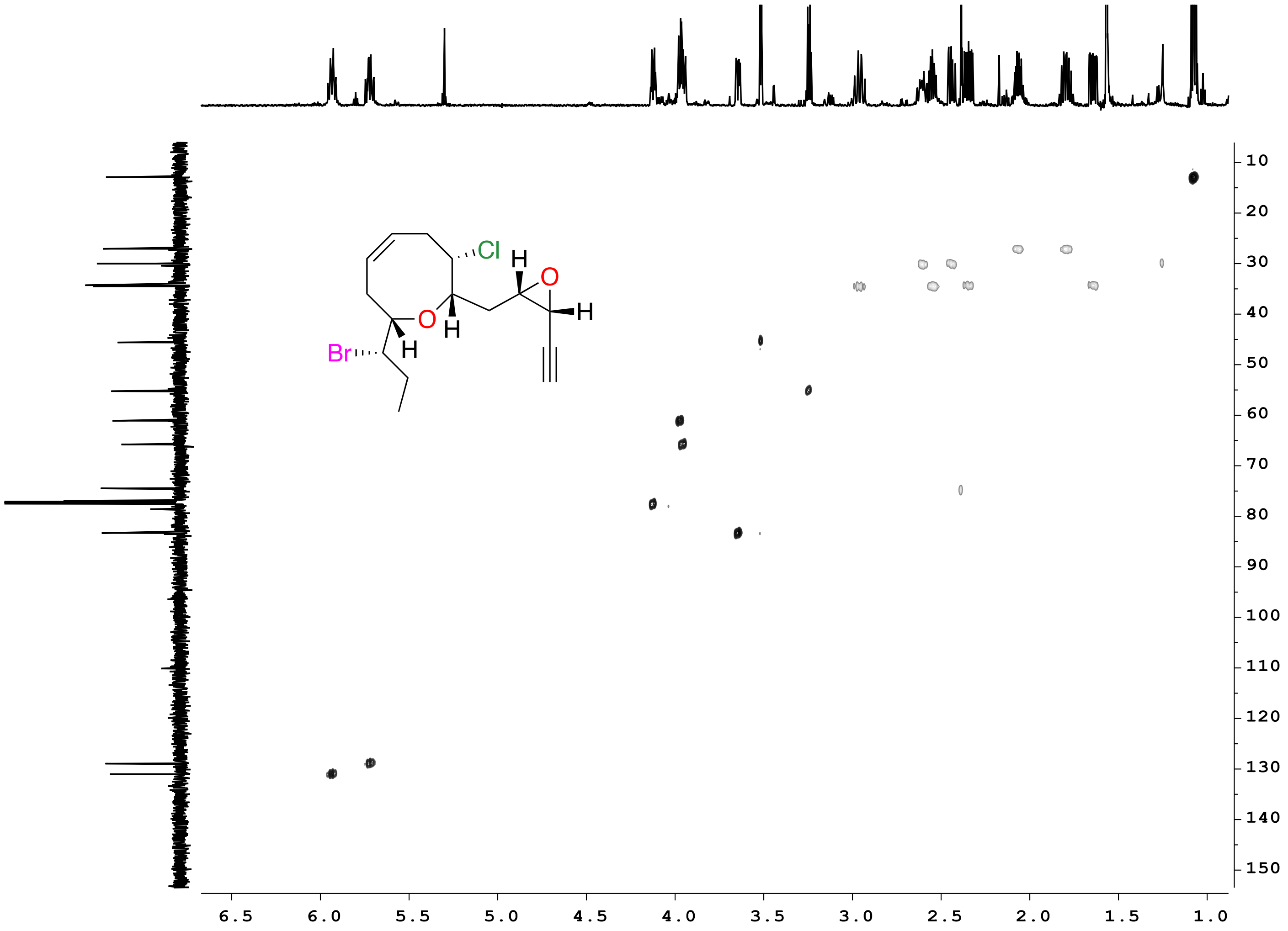

Figure S5. HMBC spectrum (600 MHz, $CDCl_3$) for (3*R*,4*S*)-epoxy-pinnatifidenyne **3**.

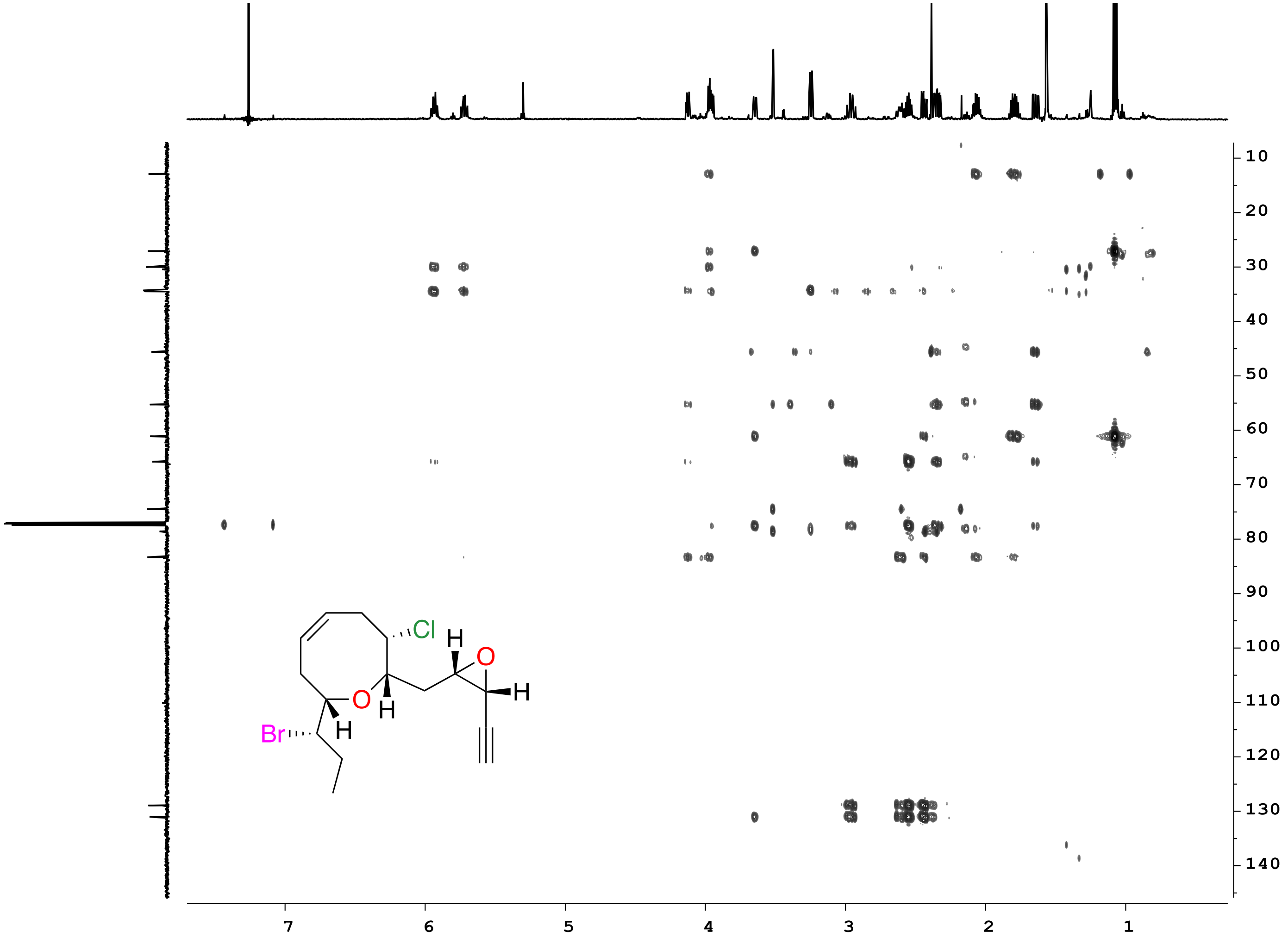

Figure S6. ROESY spectrum (600 MHz, $CDCl_3$) for (3*R*,4*S*)-epoxy-pinnatifidenyne **3**.

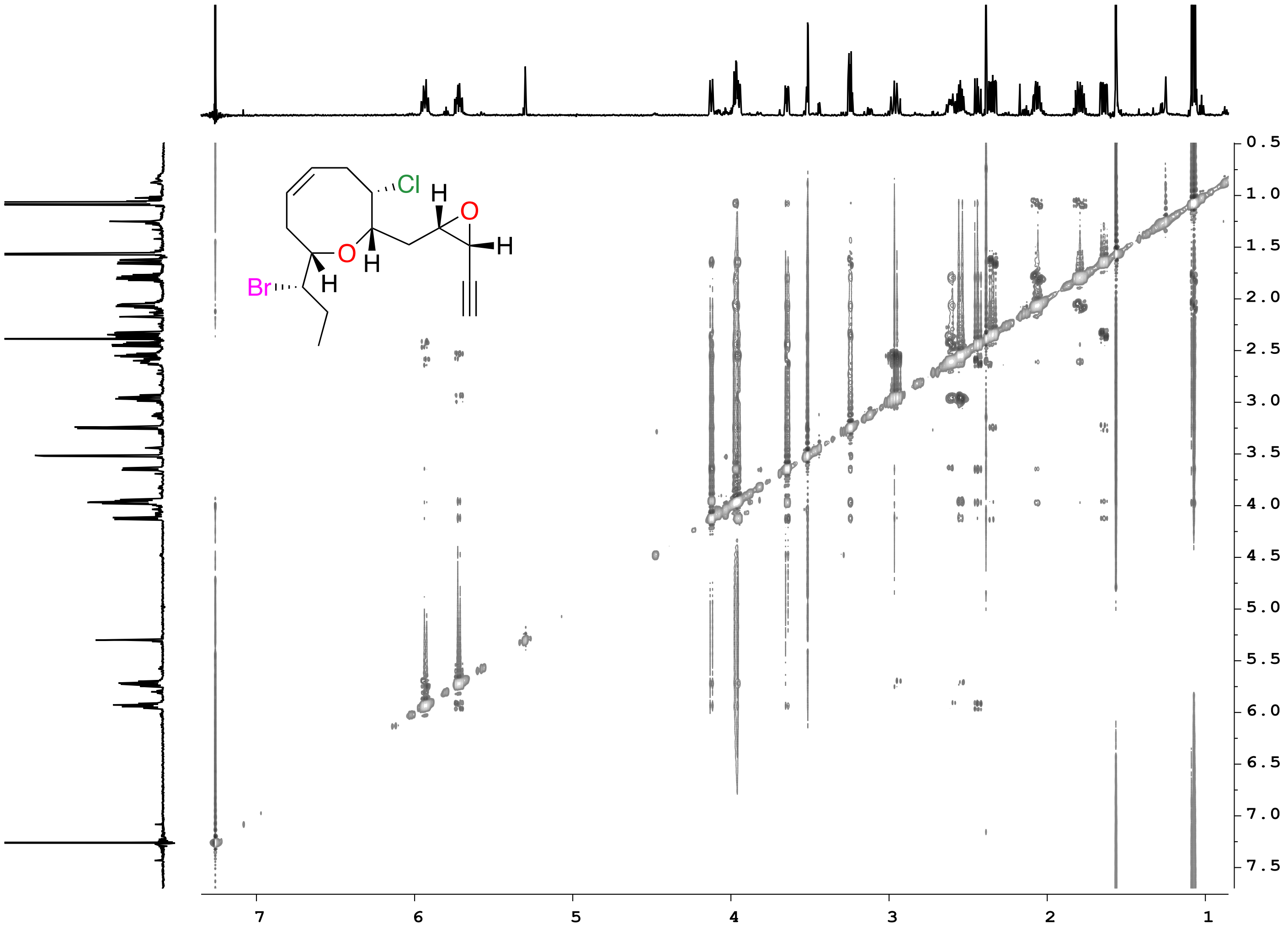

Figure S7. HSQC-HECADE spectrum (600 MHz, $CDCl_3$) for (3*R*,4*S*)-epoxy-pinnatifidenyne **3.**

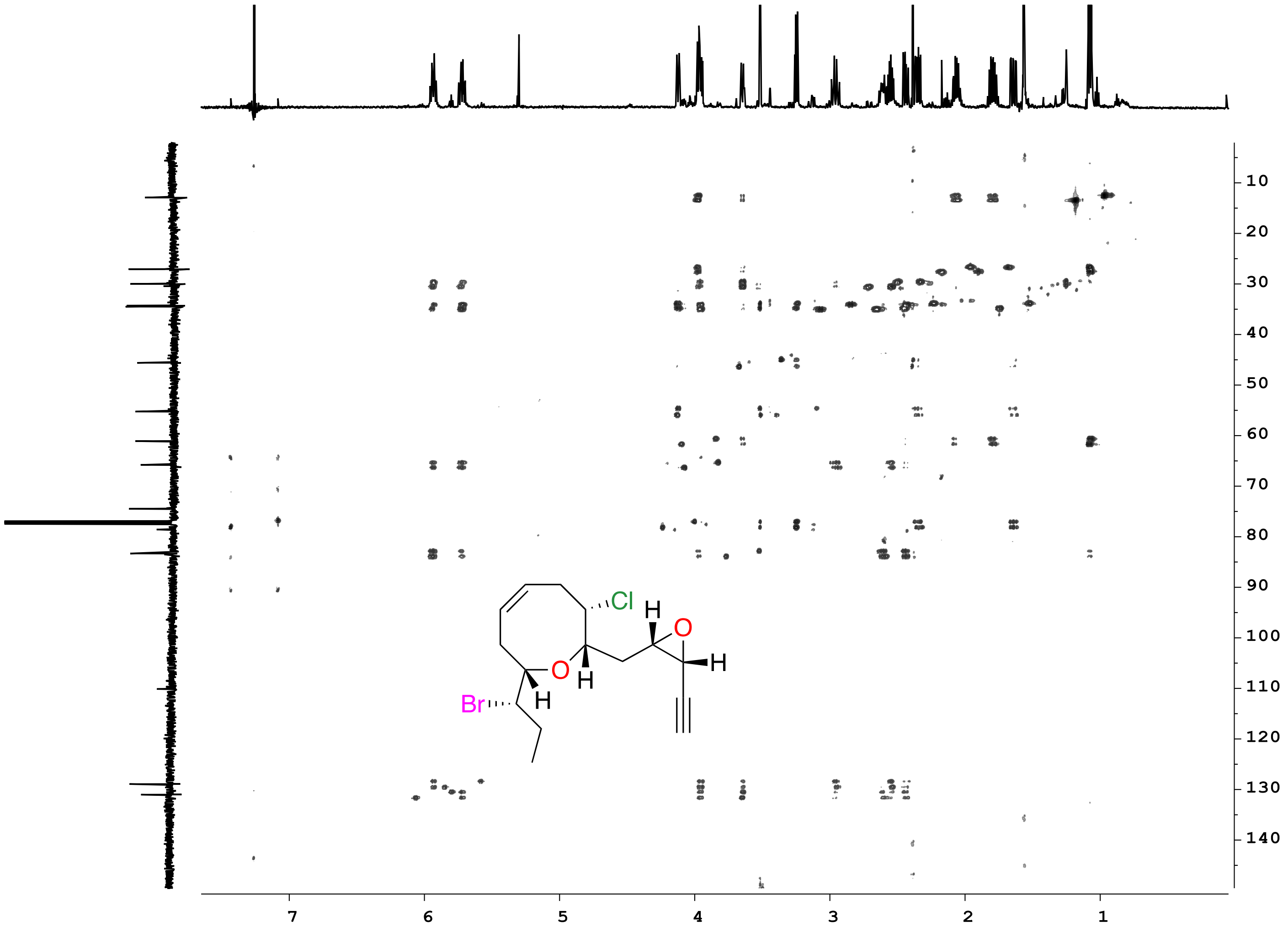

Figure S8. 1D NOESY spectra (600 MHz, $CDCl_3$) for (3*R*,4*S*)-epoxy-pinnatifidenyne **3**.

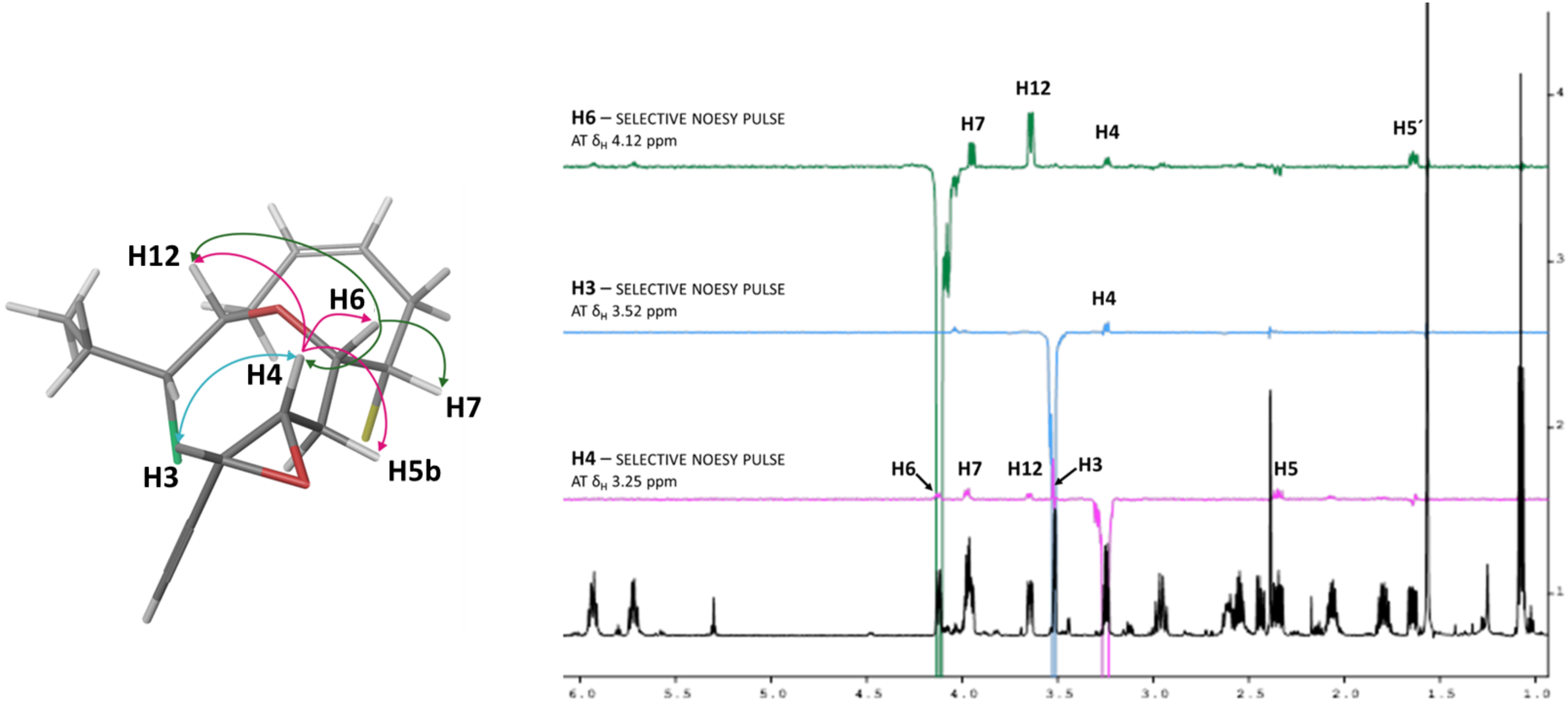

Figure S9. HRESIMS spectrum for (3*R*,4*S*)-epoxy-pinnatifidenyne **3**.

Elemental Composition Report

Multiple Mass Analysis: 2 mass(es) processed

Tolerance = 5.0 PPM / DBE: min = -10.0, max = 1000.0
Element prediction: Off
Number of isotope peaks used for i-FIT = 2

Monoisotopic Mass, Even Electron Ions
1202 formula(e) evaluated with 14 results within limits (all results (up to 1000) for each mass)
Elements Used:
C: 0-60 H: 0-120 O: 0-3 Na: 0-1 35Cl: 0-1 37Cl: 0-1 79Br: 0-1 81Br: 0-1

ESI-319 Olivia M (Omf-E) 224 (9.777) 1: TOF MS ES+ 5.28e+003

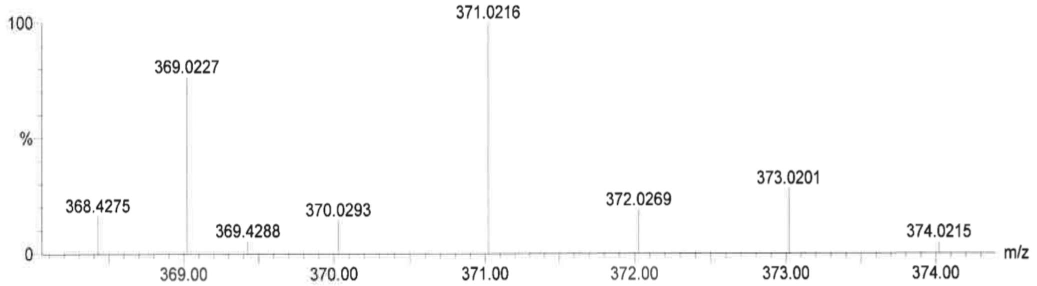

Minimum:	30.00				-10.0		
Maximum:	100.00		100.0	5.0	1000.0		

Mass	RA	Calc. Mass	mDa	PPM	DBE	i-FIT	Formula
369.0227	76.50	369.0228	-0.1	-0.3	-1.5	23.2	C12 H26 O Na 79Br 81Br
		369.0230	-0.3	-0.8	-7.5	86.9	C8 H30 O3 35Cl 79Br 81Br
		369.0233	-0.6	-1.6	4.5	2.4	C15 H20 O2 Na 35Cl 79Br
		369.0234	-0.7	-1.9	5.5	0.3	C16 H20 O Na 37Cl 81Br
		369.0236	-0.9	-2.4	-0.5	22.8	C12 H24 O3 35Cl 37Cl 81Br
		369.0239	-1.2	-3.3	11.5	7.0	C19 H14 O2 Na 35Cl 37Cl
		369.0212	1.5	4.1	-3.5	50.2	C10 H25 O3 Na 35Cl 37Cl 81Br
371.0216	100.00	371.0212	0.4	1.1	4.5	3.3	C15 H20 O2 Na 35Cl 81Br
		371.0208	0.8	2.2	18.5	68.7	C24 H11 35Cl 37Cl
		371.0227	-1.1	-3.0	7.5	0.6	C17 H19 O2 37Cl 79Br
		371.0203	1.3	3.5	4.5	3.5	C15 H20 O2 Na 37Cl 79Br
		371.0202	1.4	3.8	11.5	16.8	C20 H17 35Cl 79Br
		371.0200	1.6	4.3	-7.5	114.8	C8 H30 O3 37Cl 79Br 81Br
		371.0234	-1.8	-4.9	13.5	26.7	C21 H15 Na 81Br

Figure S10. ^{1}H-NMR spectrum (600 MHz, $CDCl_3$) for (3*S*,4*R*)-epoxy-pinnatifidenyne **4**.

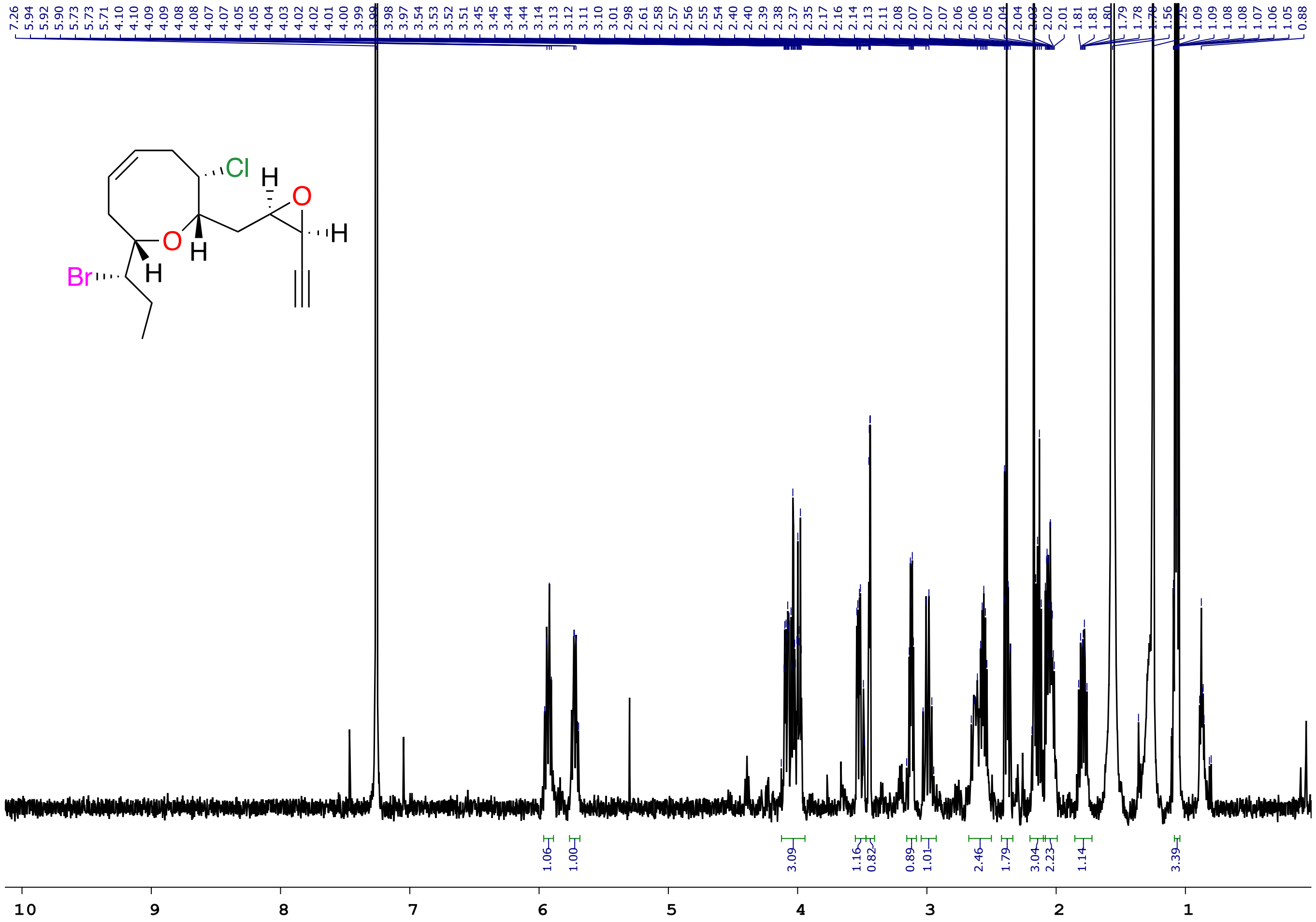

Figure S11. ^{13}C-NMR spectrum (150 MHz, $CDCl_3$) for (3*S*,4*R*)-epoxy-pinnatifidenyne **4**.

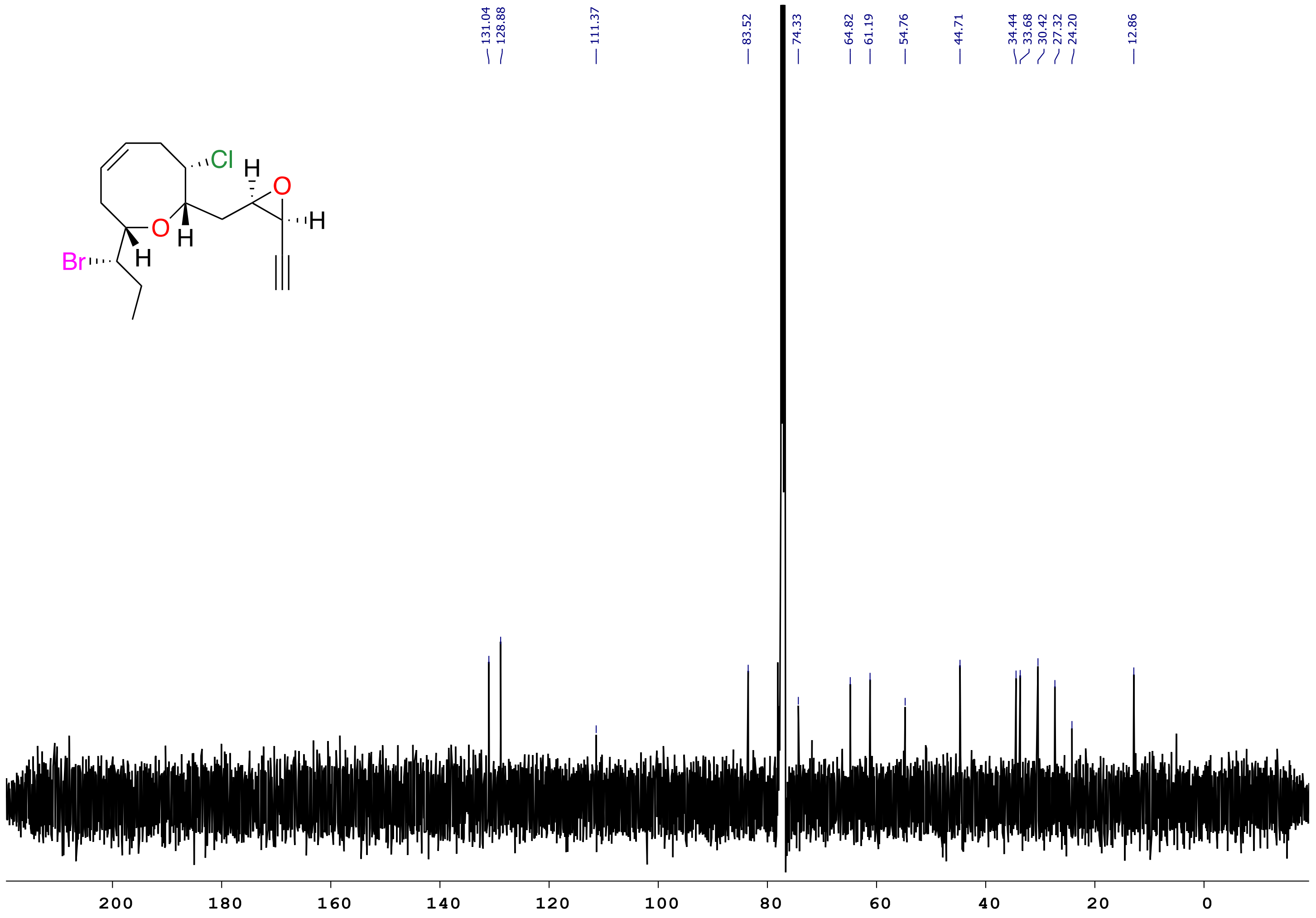

Figure S12. HSQCed spectrum (600 MHz, $CDCl_3$) for (3*S*,4*R*)-epoxy-pinnatifidenyne **4**.

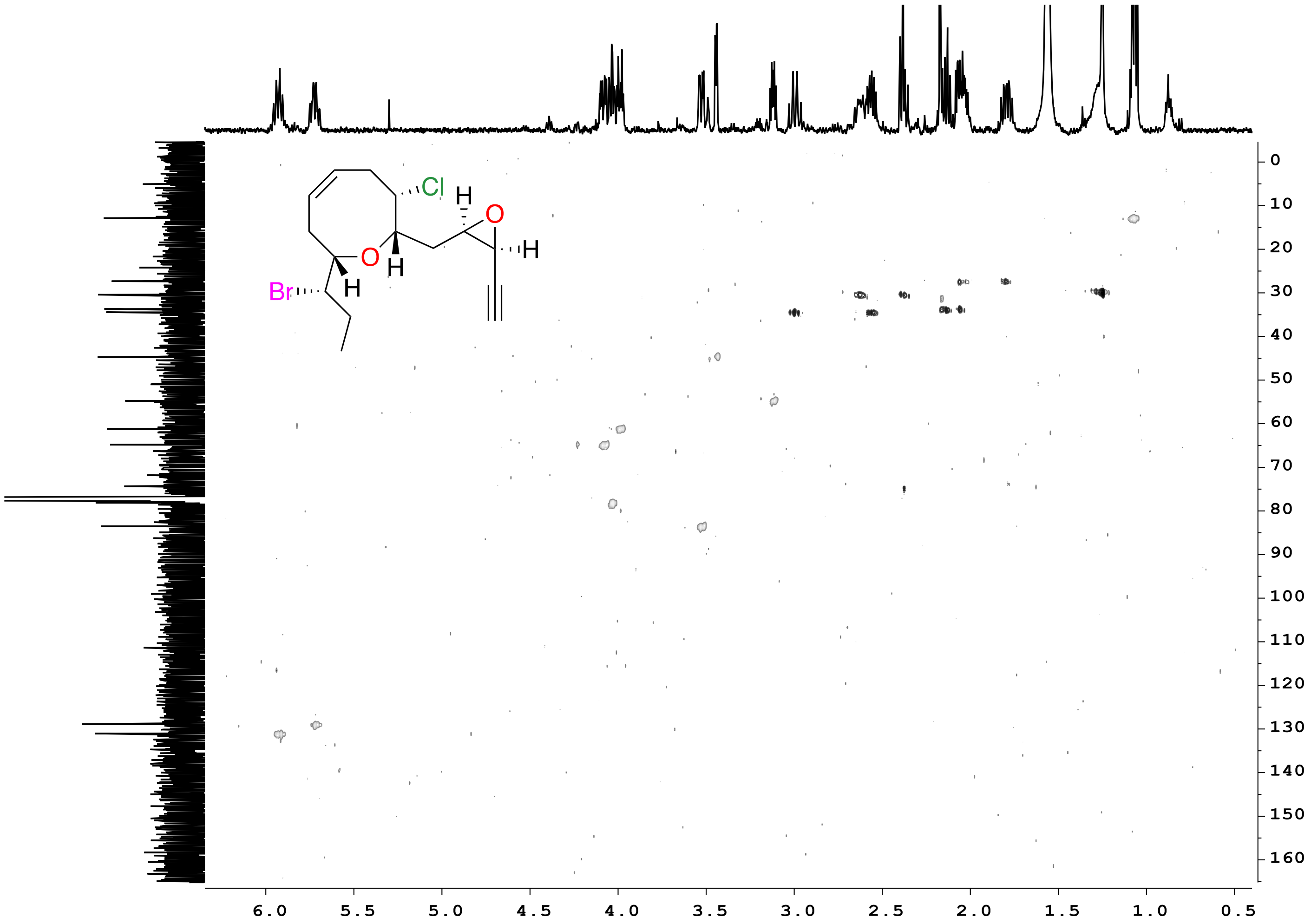

Figure S13. COSY ^{1}H-^{1}H spectrum (600 MHz, $CDCl_3$) for (3*S*,4*R*)-epoxy-pinnatifidenyne **4**.

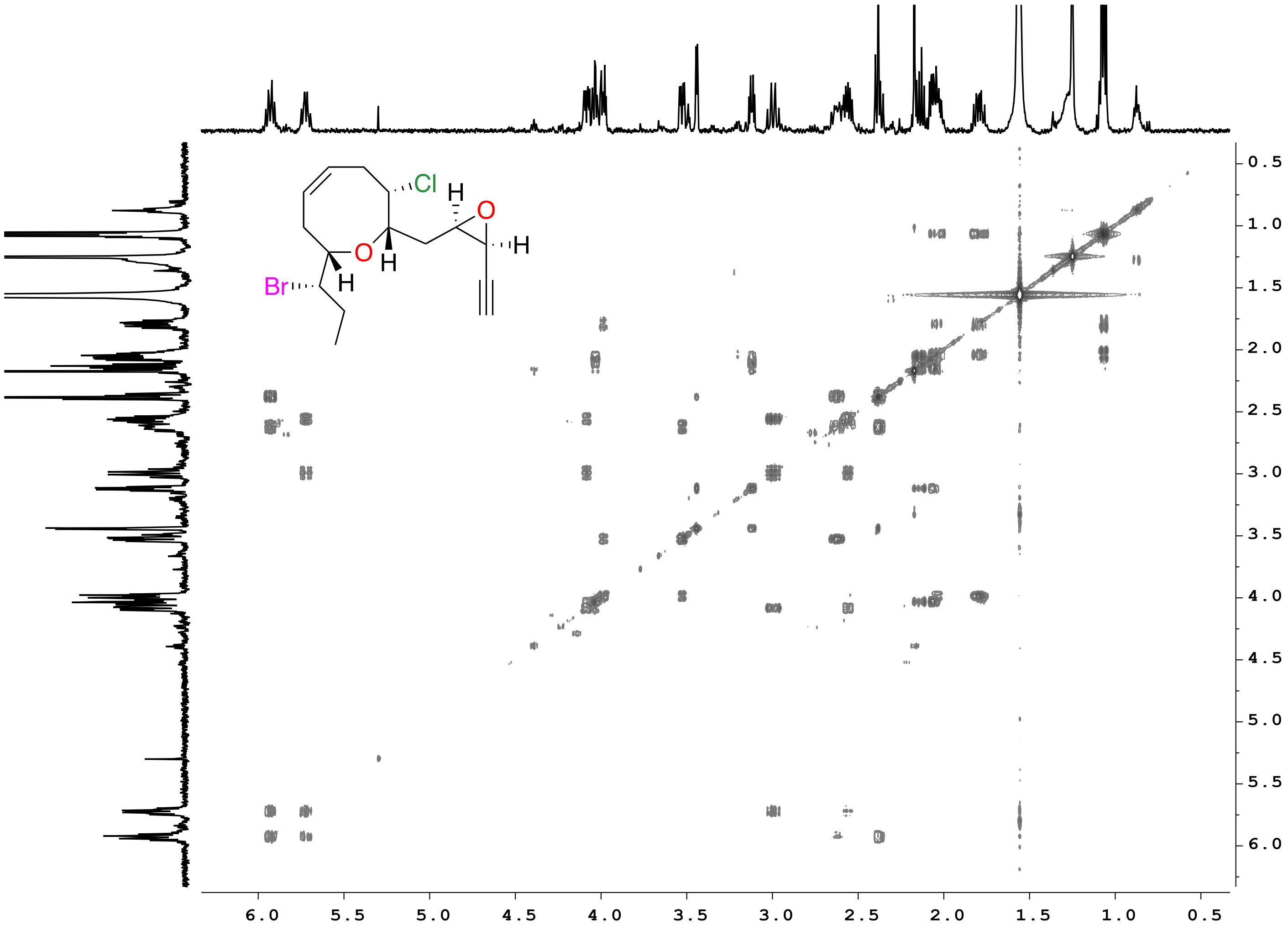

Figure S14. HMBC spectrum (600 MHz, $CDCl_3$) for (3*S*,4*R*)-epoxy-pinnatifidenyne **4**.

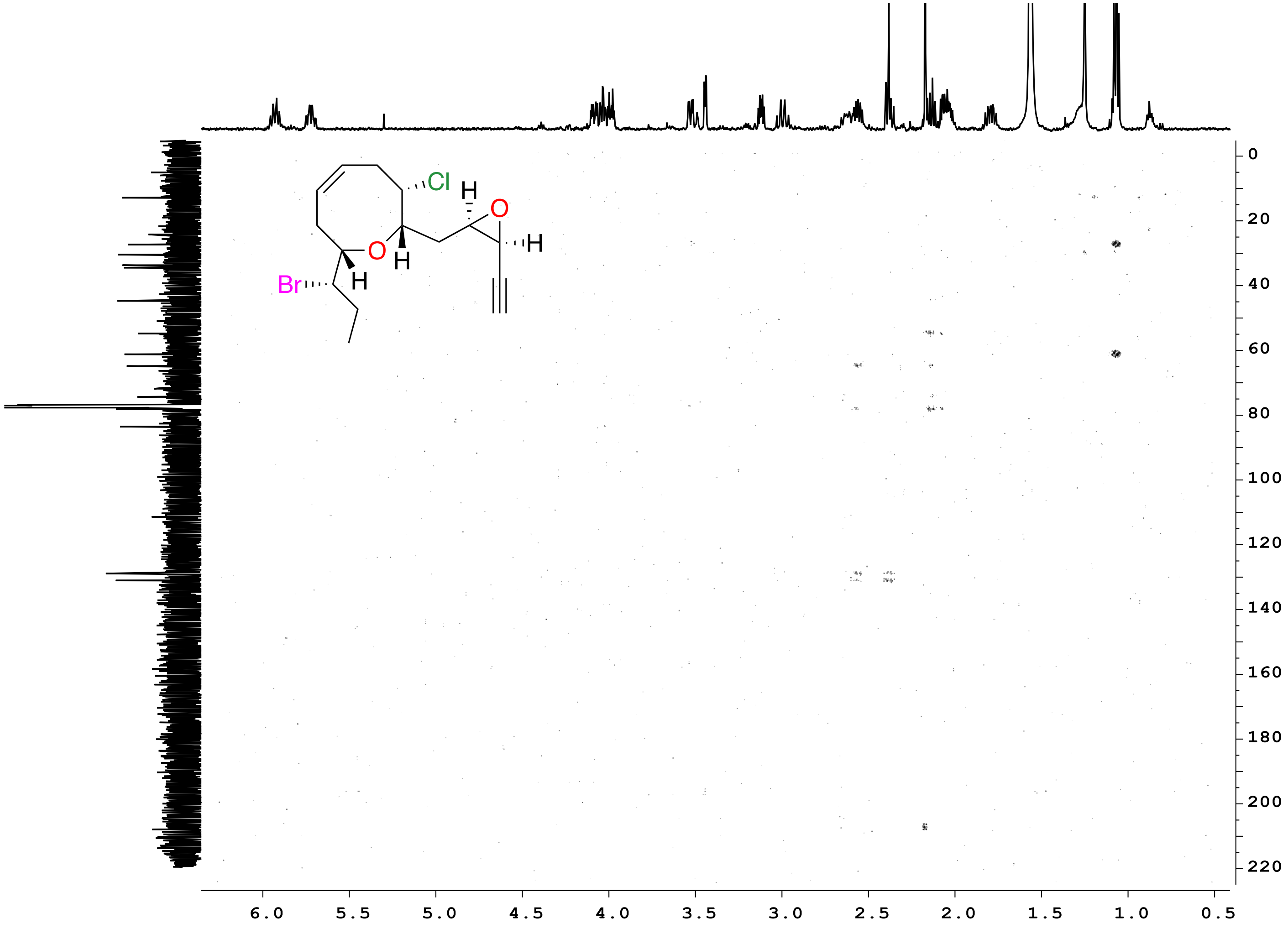

Figure S15. ROESY spectrum (600 MHz, $CDCl_3$) for (3*S*,4*R*)-epoxy-pinnatifidenyne **4**.

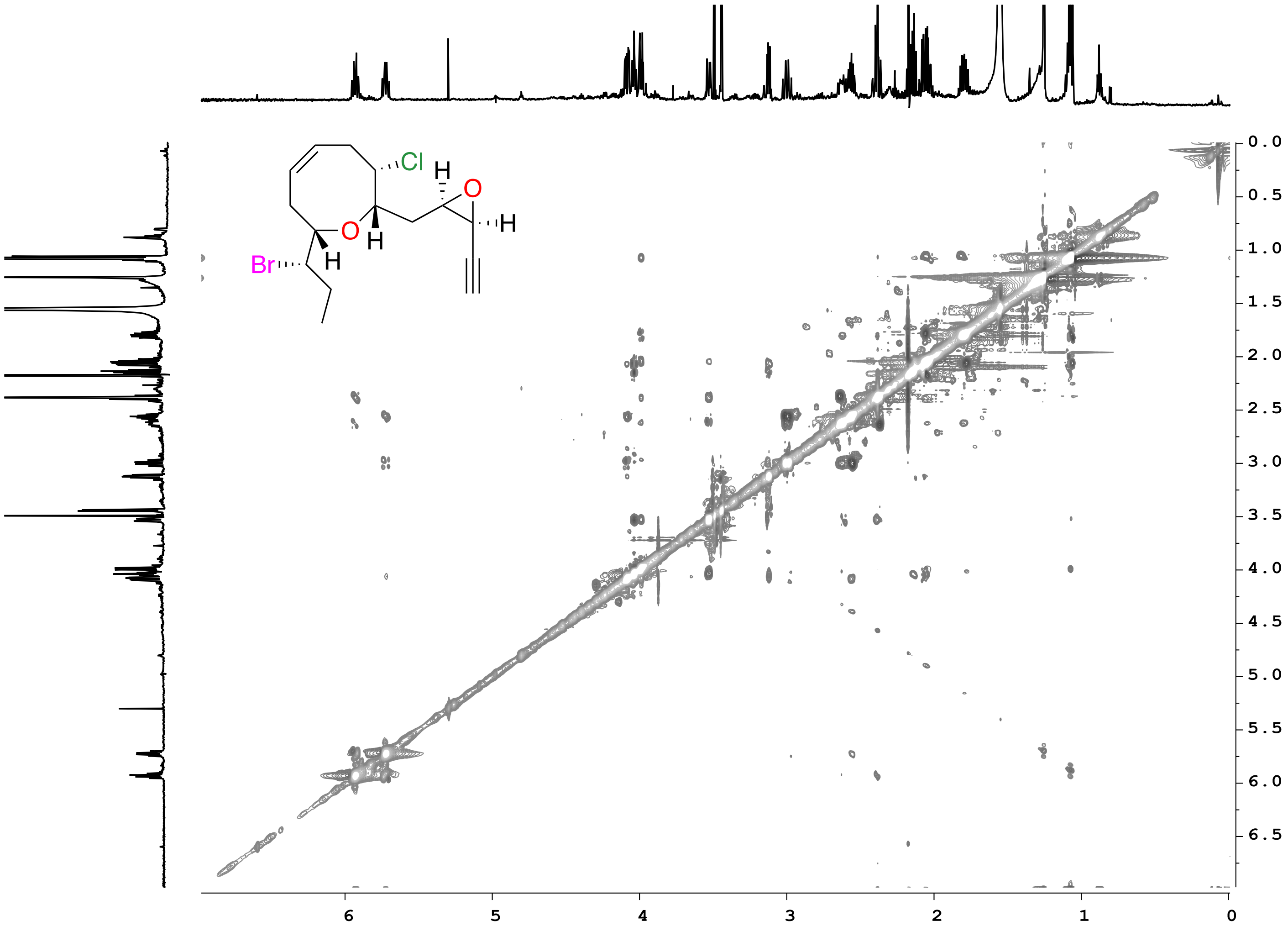

Figure S16. 1D NOESY spectra (600 MHz, $CDCl_3$) for (3*S*,4*R*)-epoxy-pinnatifidenyne **4**.

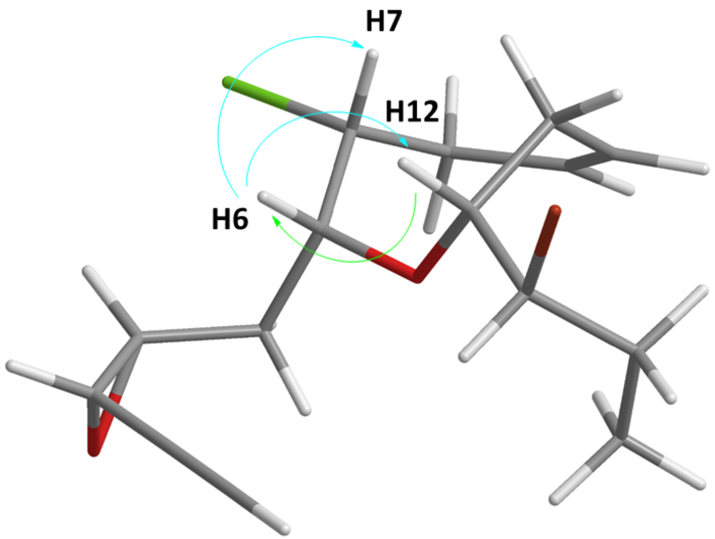

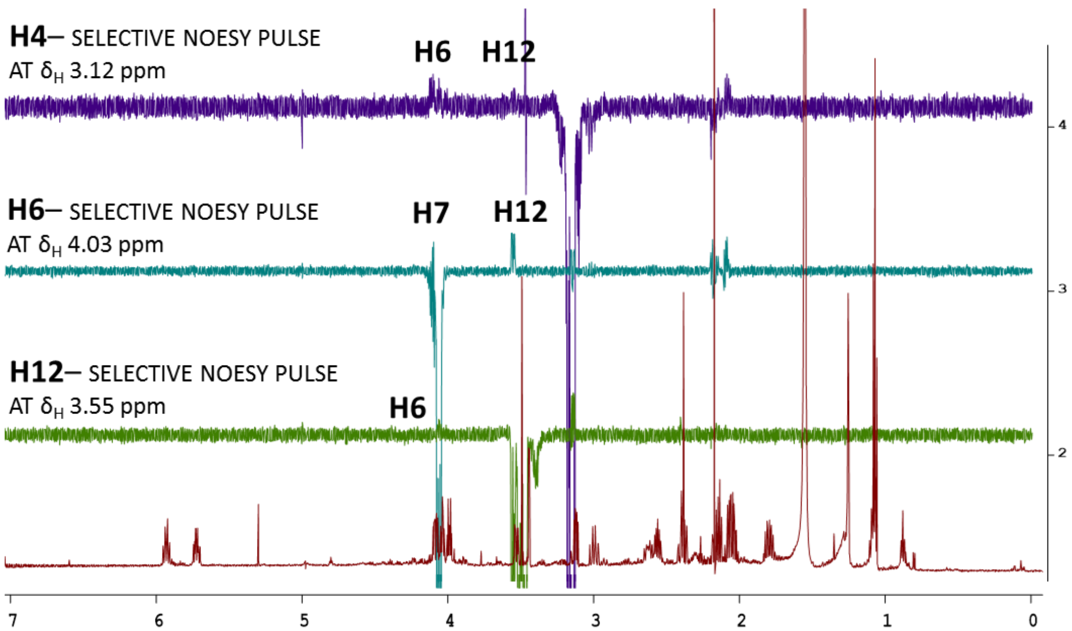

Figure S17. HRESIMS spectrum for (3*S*,4*R*)-epoxy-pinnatifidenyne **4**.

Elemental Composition Report

Multiple Mass Analysis: 4 mass(es) processed
Tolerance = 5.0 PPM / DBE: min = -10.0, max = 1000.0
Element prediction: Off
Number of isotope peaks used for i-FIT = 2

Monoisotopic Mass, Even Electron Ions
625 formula(e) evaluated with 11 results within limits (all results (up to 1000) for each mass)
Elements Used:
C: 0-20 H: 0-25 O: 0-2 Na: 0-1 35Cl: 0-1 37Cl: 0-1 79Br: 0-1 81Br: 0-1

(ESI 17-784) Adrian M (AM Epoxido B) 34 (1.487)

1: TOF MS ES+
6.73e+003

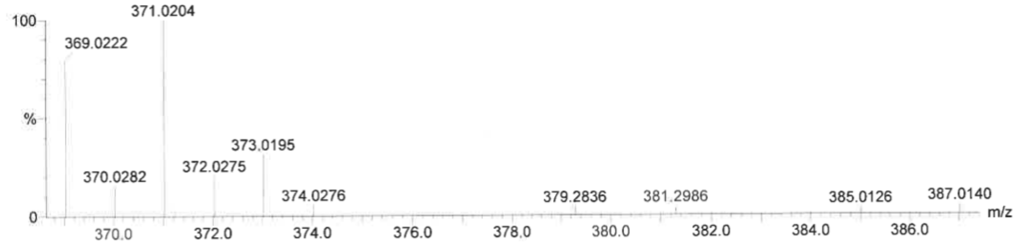

Minimum:	20.00				-10.0		
Maximum:	100.00		100.0	5.0	1000.0		
Mass	RA	Calc. Mass	mDa	PPM	DBE	i-FIT	Formula
369.0222	79.43	369.0233	-1.1	-3.0	4.5	8.4	C15 H20 O2 Na 35Cl 79Br
		369.0234	-1.2	-3.3	5.5	3.0	C16 H20 O Na 37Cl 81Br
		369.0239	-1.7	-4.6	11.5	3.6	C19 H14 O2 Na 35Cl 37Cl
371.0204	100.00	371.0203	0.1	0.3	4.5	18.3	C15 H20 O2 Na 37Cl 79Br
		371.0202	0.2	0.5	11.5	5.2	C20 H17 35Cl 79Br
		371.0212	-0.8	-2.2	4.5	18.2	C15 H20 O2 Na 35Cl 81Br
372.0275	20.59	---					
373.0195	31.36	373.0201	-0.6	-1.6	0.5	4.2	C13 H25 O2 79Br 81Br
		373.0204	-0.9	-2.4	12.5	11.0	C20 H15 O Na 79Br
		373.0183	1.2	3.2	4.5	0.5	C15 H20 O2 Na 37Cl 81Br
		373.0207	-1.2	-3.2	7.5	1.4	C17 H19 O2 37Cl 81Br
		373.0182	1.3	3.5	11.5	11.2	C20 H17 35Cl 81Br

Figure S18. ^{1}H-NMR spectrum (600 MHz, $CDCl_3$) for (9*R*,10*S*)-epoxy-*Z*-pinnatifidenyne **5**.

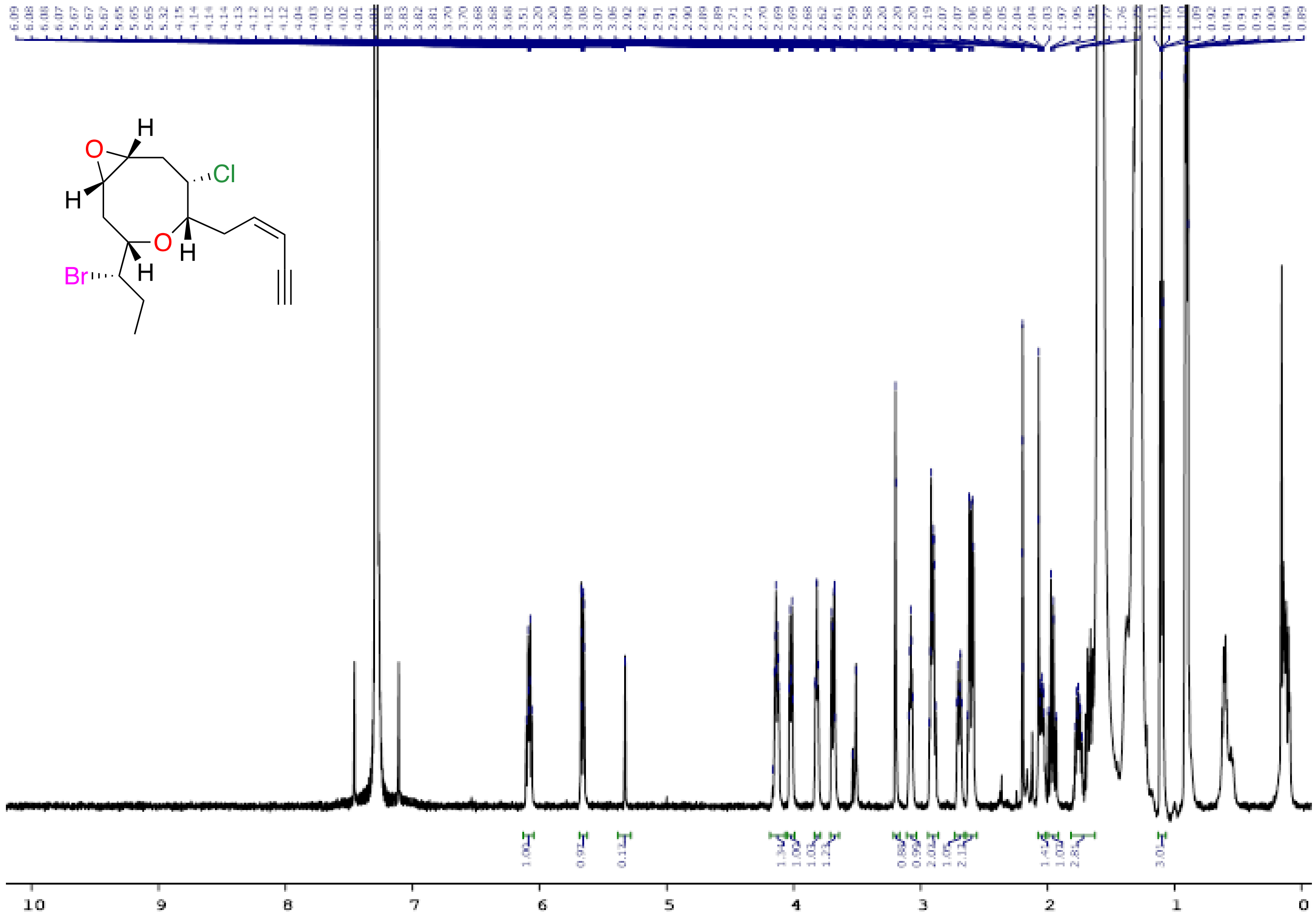

Figure S19. HSQCed spectrum (600 MHz, $CDCl_3$) for (9*R*,10*S*)-epoxy-*Z*-pinnatifidenyne **5**.

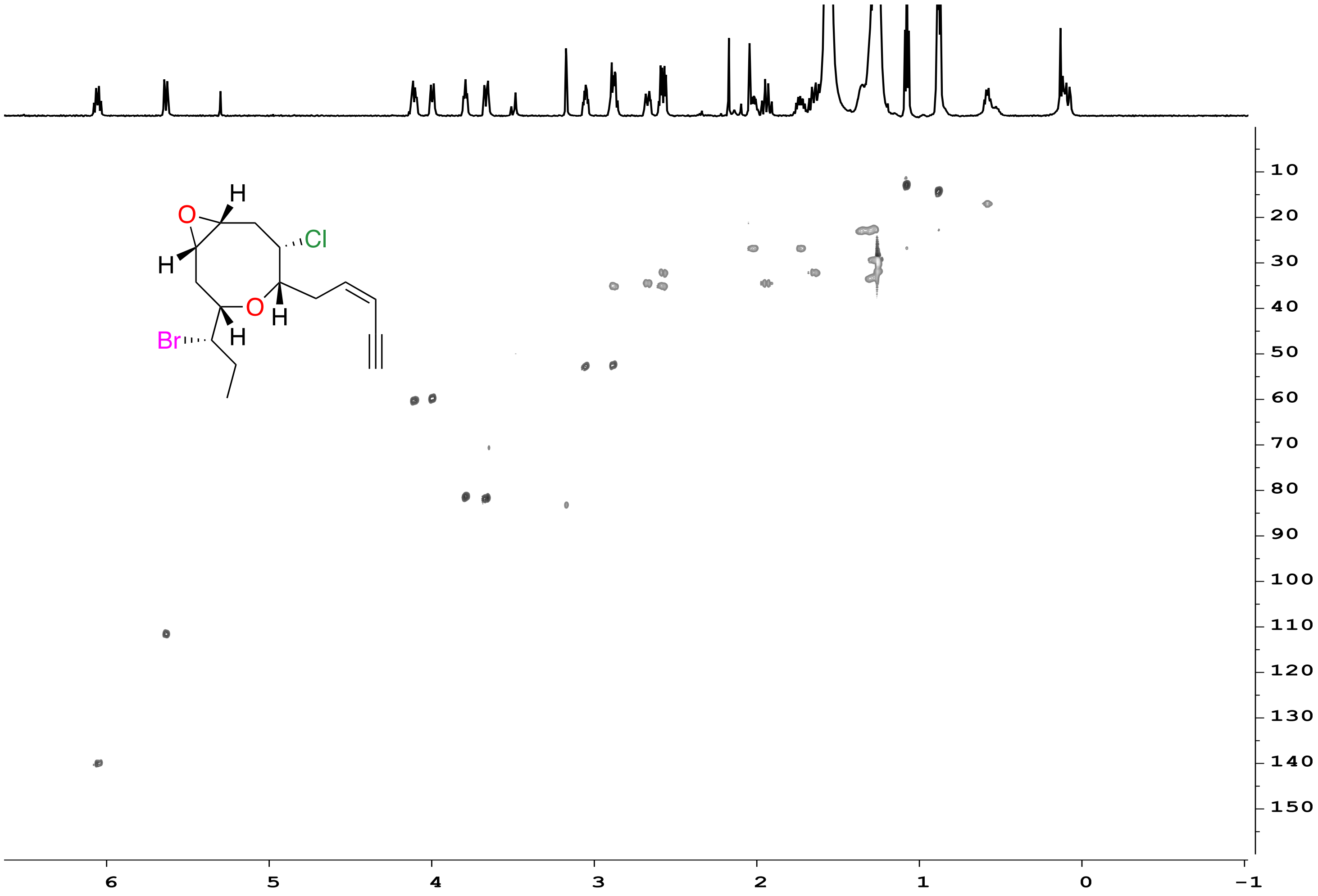

Figure S20. COSY ^1H-^1H spectrum (600 MHz, $CDCl_3$) for (9*R*,10*S*)-epoxy-*Z*-pinnatifidenyne **5**.

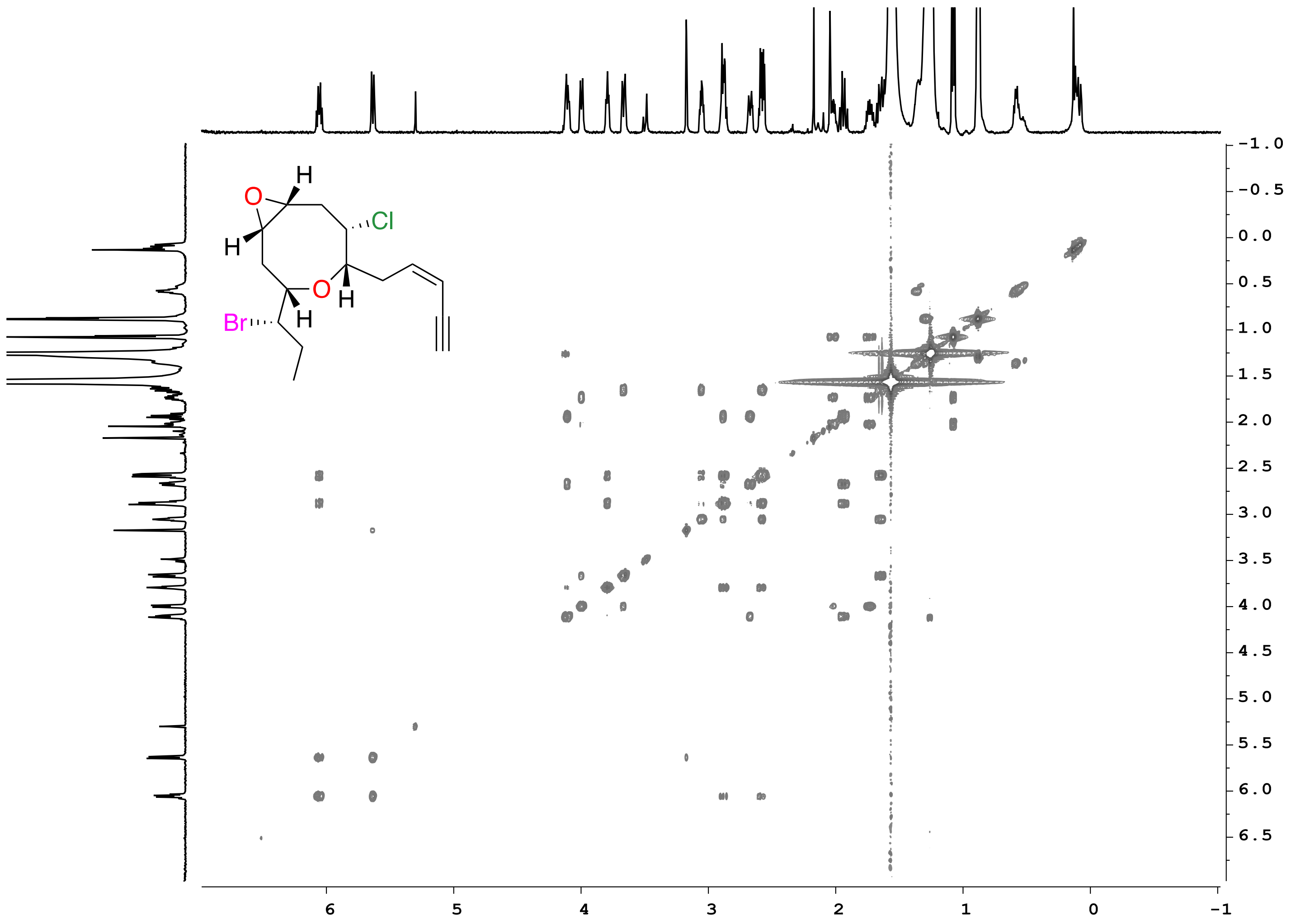

Figure S21. HMBC spectrum (600 MHz, $CDCl_3$) for (9*R*,10*S*)-epoxy-*Z*-pinnatifidenyne **5**.

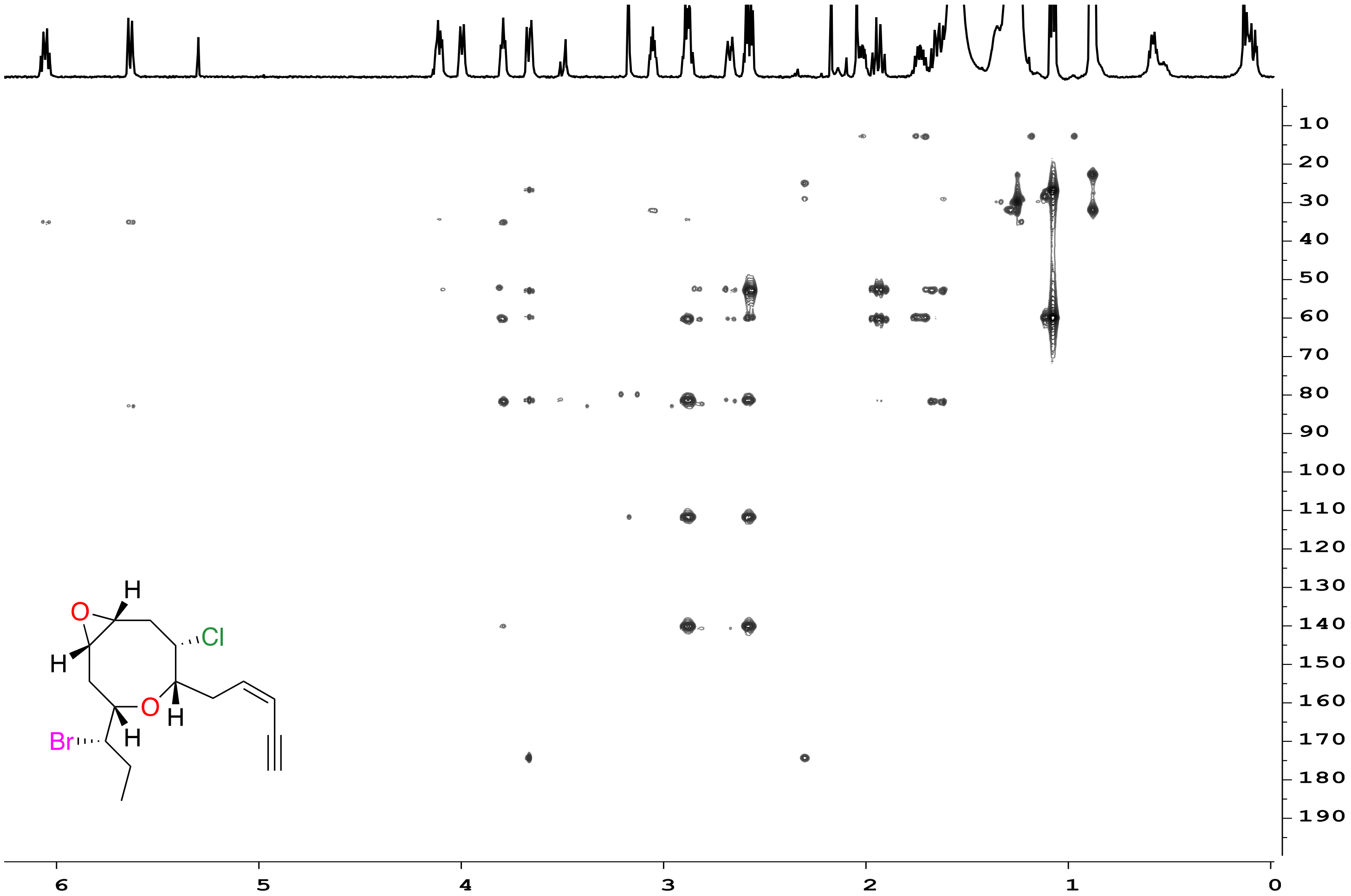

Figure S22. ROESY spectrum (600 MHz, $CDCl_3$) for (9*R*,10*S*)-epoxy-*Z*-pinnatifidenyne **5**.

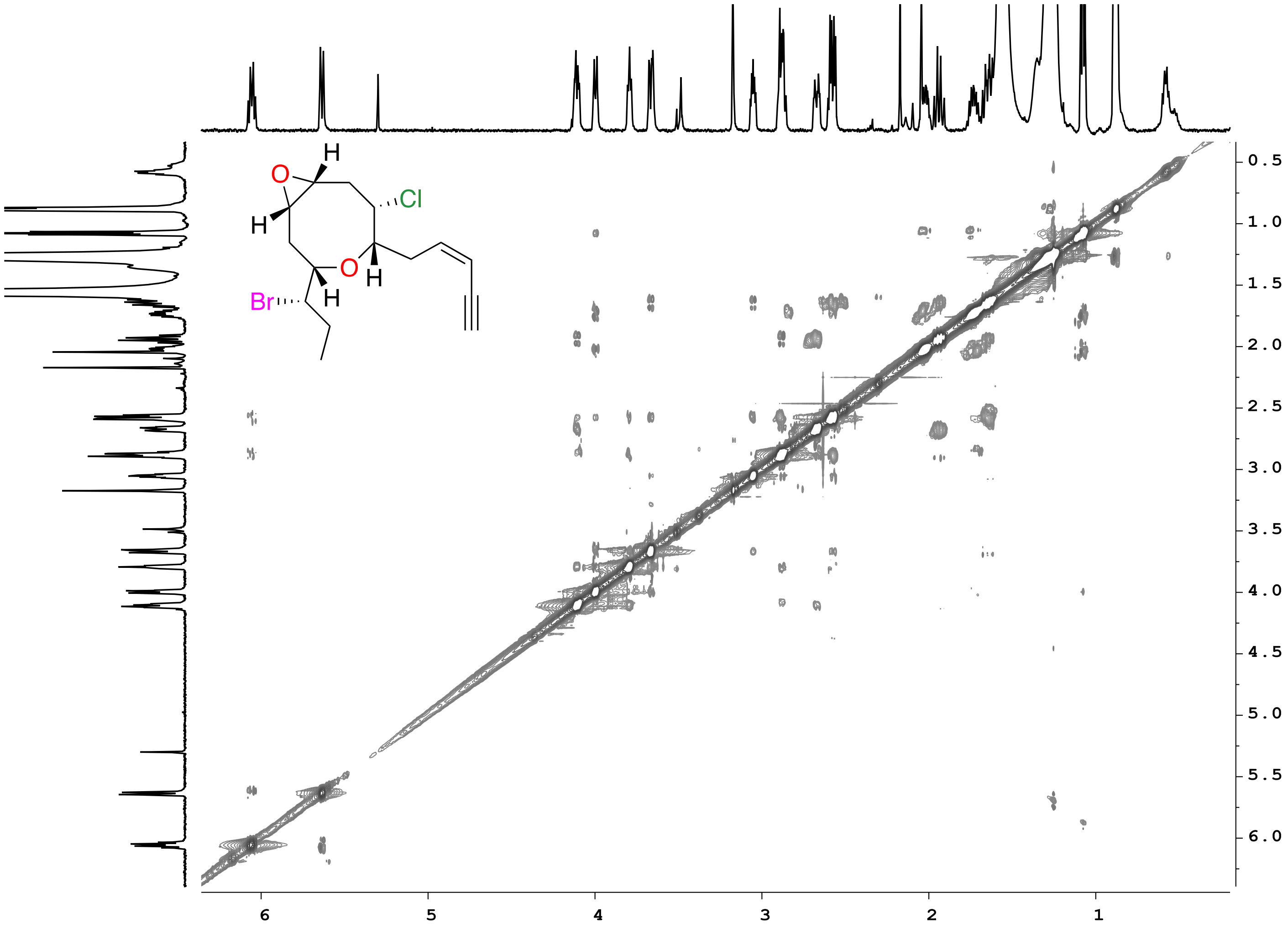

Figure S23. 1D NOESY spectra (600 MHz, $CDCl_3$) for (9*R*,10*S*)-epoxy-*Z*-pinnatifidenyne **5**.

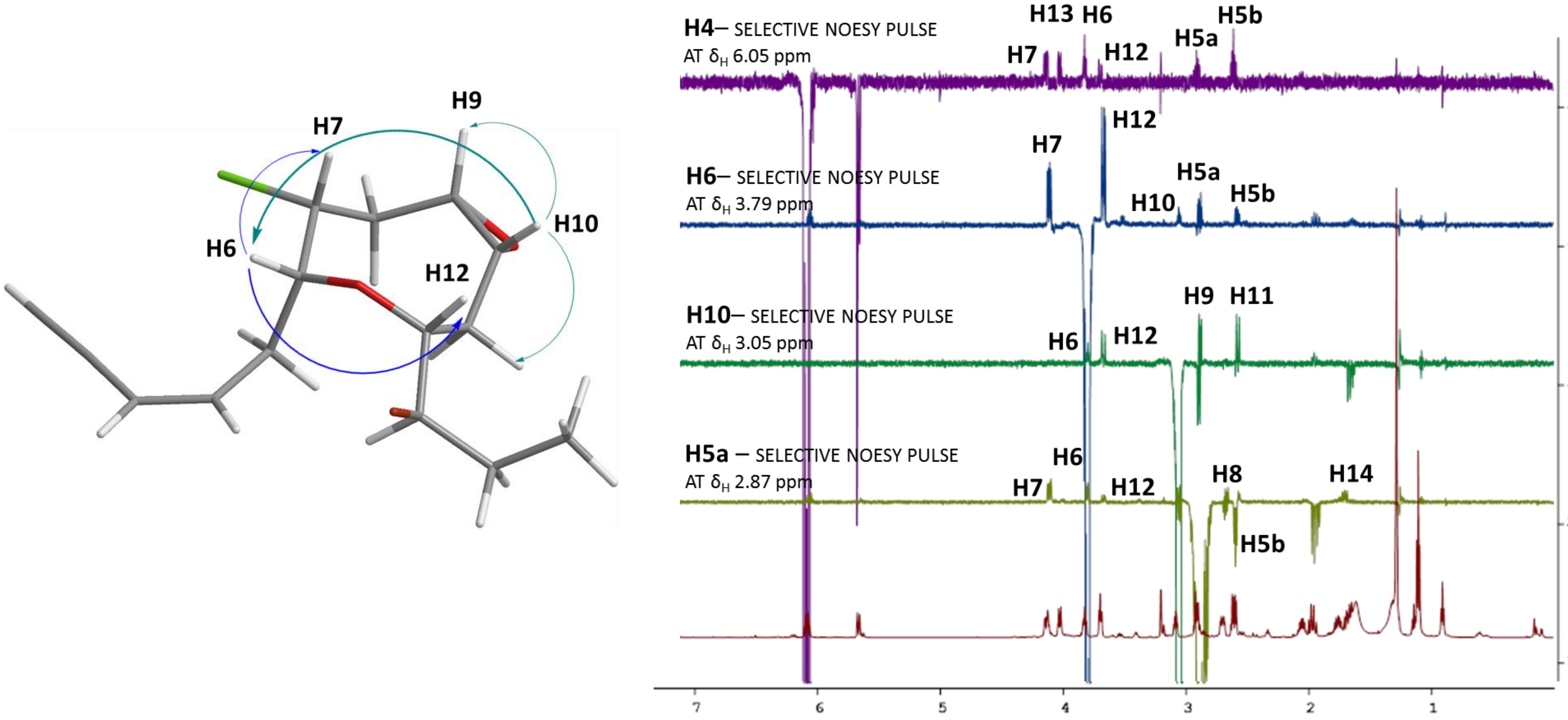

Figure S24. HRESIMS spectrum for (9*R*,10*S*)-epoxy-*Z*-pinnatifidenyne **5**.

Elemental Composition Report

Page

Multiple Mass Analysis: 3 mass(es) processed

Tolerance = 5.0 PPM / DBE: min = -10.0, max = 1000.0
Element prediction: Off
Number of isotope peaks used for i-FIT = 2

Monoisotopic Mass, Even Electron Ions
469 formula(e) evaluated with 12 results within limits (all results (up to 1000) for each mass)
Elements Used:
C: 0-20 H: 0-25 O: 0-2 Na: 0-1 35Cl: 0-1 37Cl: 0-1 79Br: 0-1 81Br: 0-1

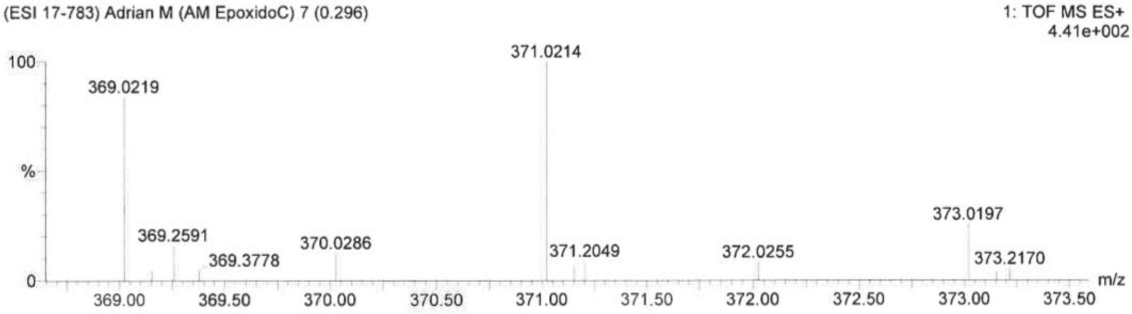

Mass	RA	Calc. Mass	mDa	PPM	DBE	i-FIT	Formula
Minimum:	20.00				-10.0		
Maximum:	100.00		100.0	5.0	1000.0		
369.0219	83.92	369.0233	-1.4	-3.8	4.5	0.5	C15 H20 O2 Na 35Cl 79Br
		369.0234	-1.5	-4.1	5.5	1.0	C16 H20 O Na 37Cl 81Br
		369.0202	1.7	4.6	3.5	0.5	C15 H22 O 35Cl 37Cl 79Br
371.0214	100.00	371.0212	0.2	0.5	4.5	14.7	C15 H20 O2 Na 35Cl 81Br
		371.0203	1.1	3.0	4.5	14.7	C15 H20 O2 Na 37Cl 79Br
		371.0202	1.2	3.2	11.5	32.7	C20 H17 35Cl 79Br
		371.0227	-1.3	-3.5	7.5	21.7	C17 H19 O2 37Cl 79Br
373.0197	25.40	373.0201	-0.4	-1.1	0.5	n/a	C13 H25 O2 79Br 81Br
		373.0204	-0.7	-1.9	12.5	n/a	C20 H15 O Na 79Br
		373.0207	-1.0	-2.7	7.5	n/a	C17 H19 O2 37Cl 81Br
		373.0183	1.4	3.8	4.5	n/a	C15 H20 O2 Na 37Cl 81Br
		373.0182	1.5	4.0	11.5	n/a	C20 H17 35Cl 81Br

Figure S25. ^{1}H-NMR spectrum (600 MHz, $CDCl_3$) for pinnatifidehyde **6**.

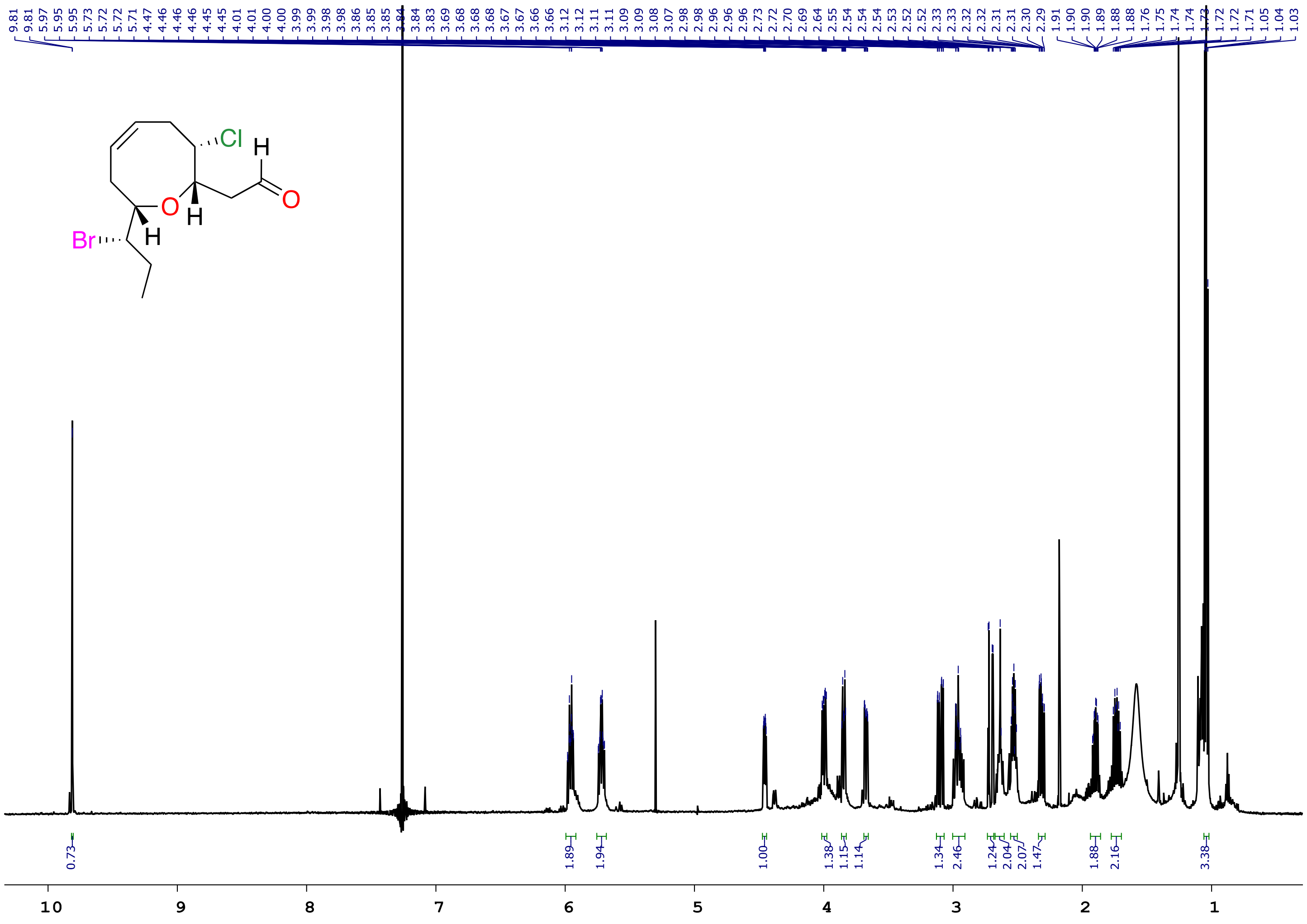

Figure S26. ^{13}C-NMR spectrum (125 MHz, $CDCl_3$) for pinnatifidehyde **6**.

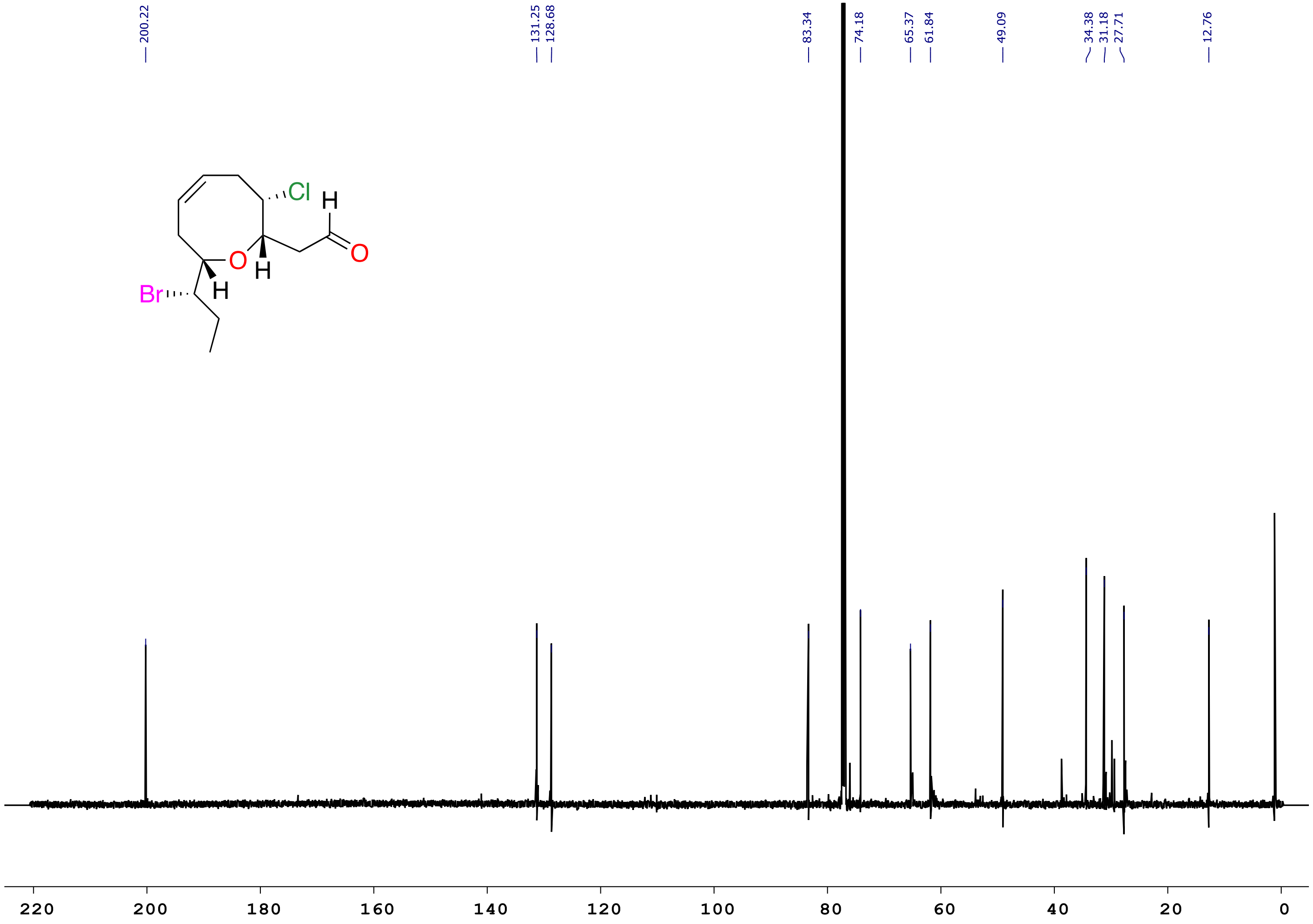

Figure S27. COSY ^{1}H-^{1}H spectrum (600 MHz, $CDCl_3$) for pinnatifidehyde **6**.

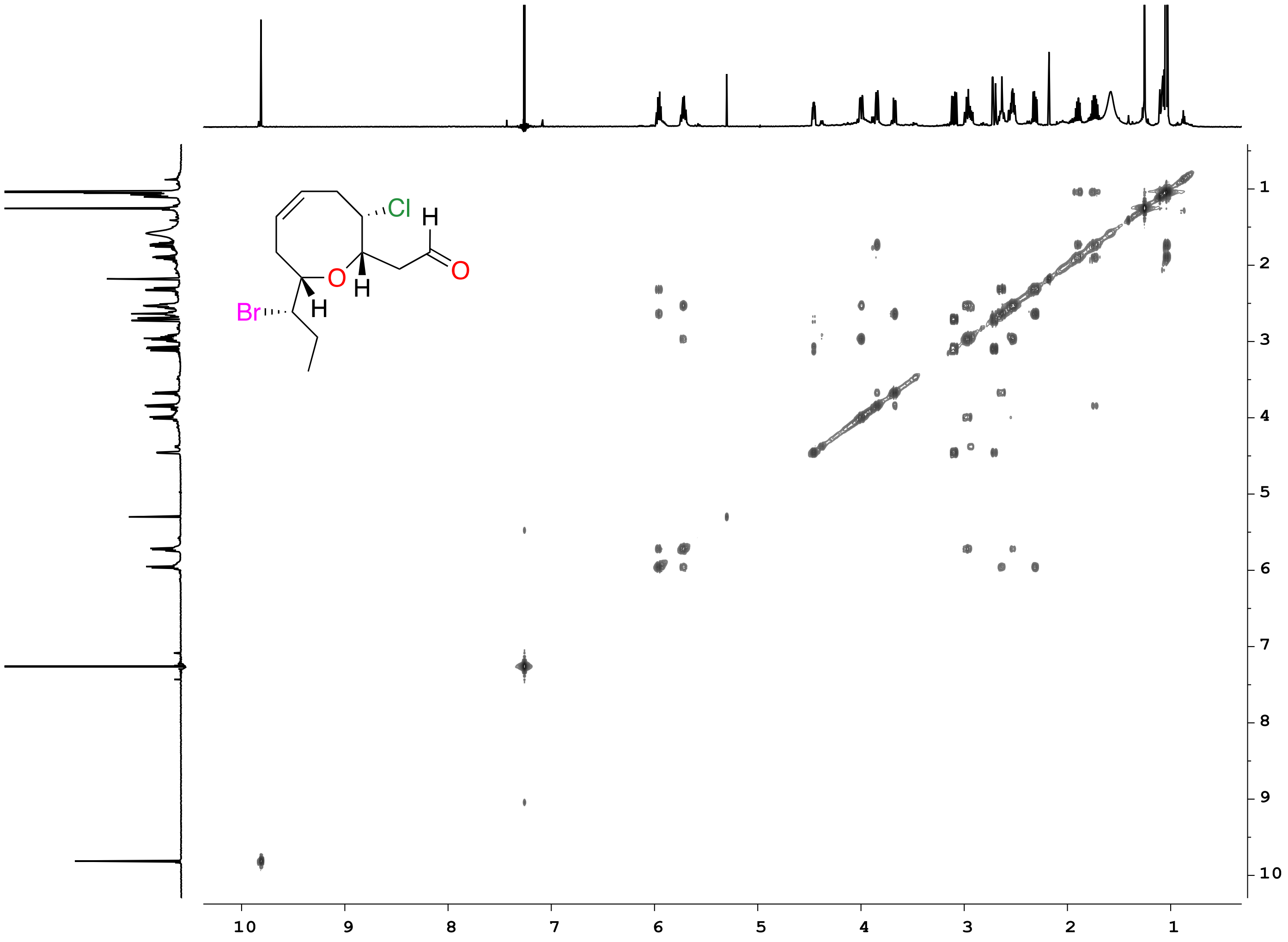

Figure S28. HSQC spectrum (600 MHz, $CDCl_3$) for pinnatifidehyde **6**.

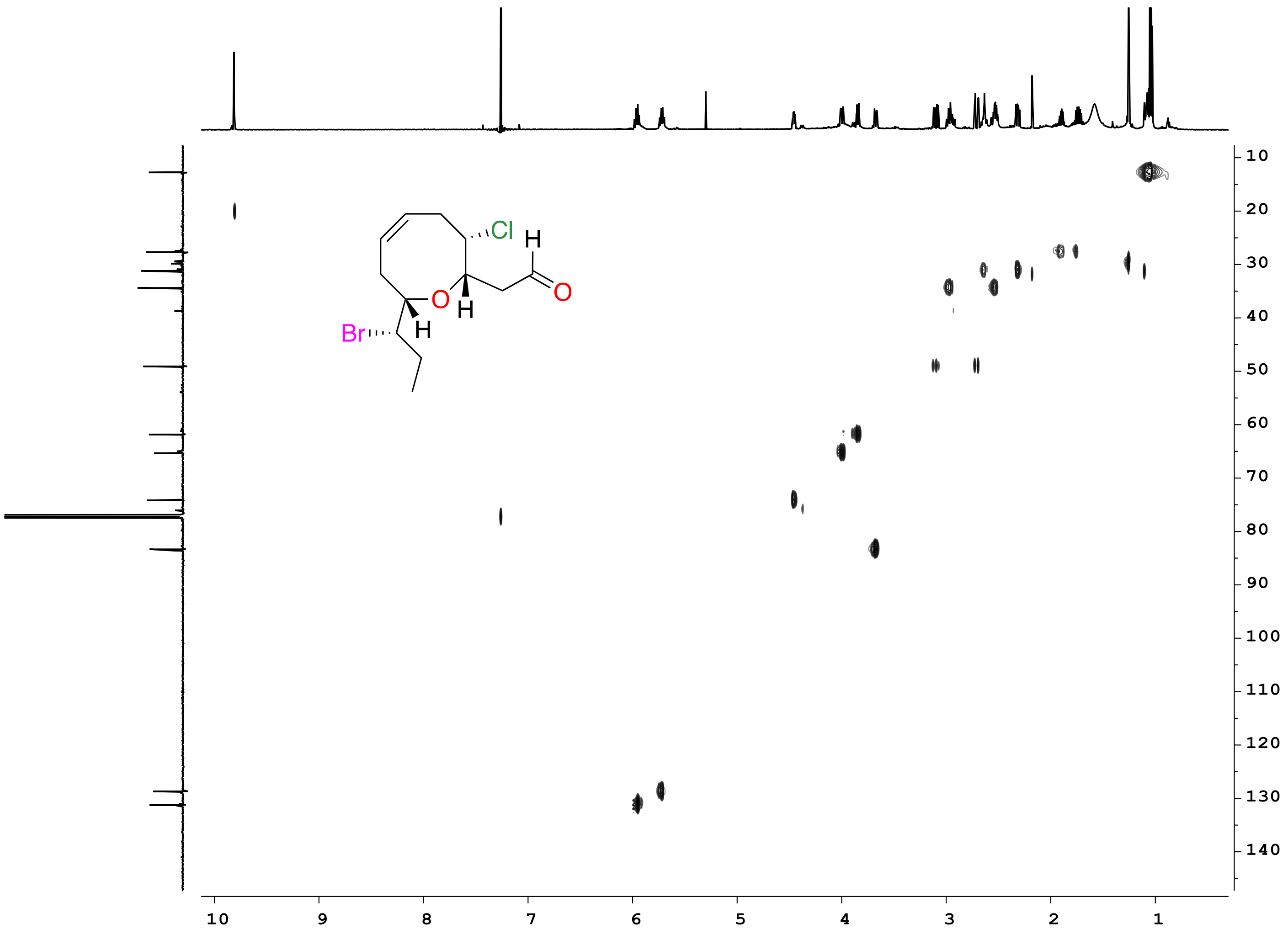

Figure S29. HMBC spectrum (600 MHz, $CDCl_3$) for pinnatifidehyde **6**.

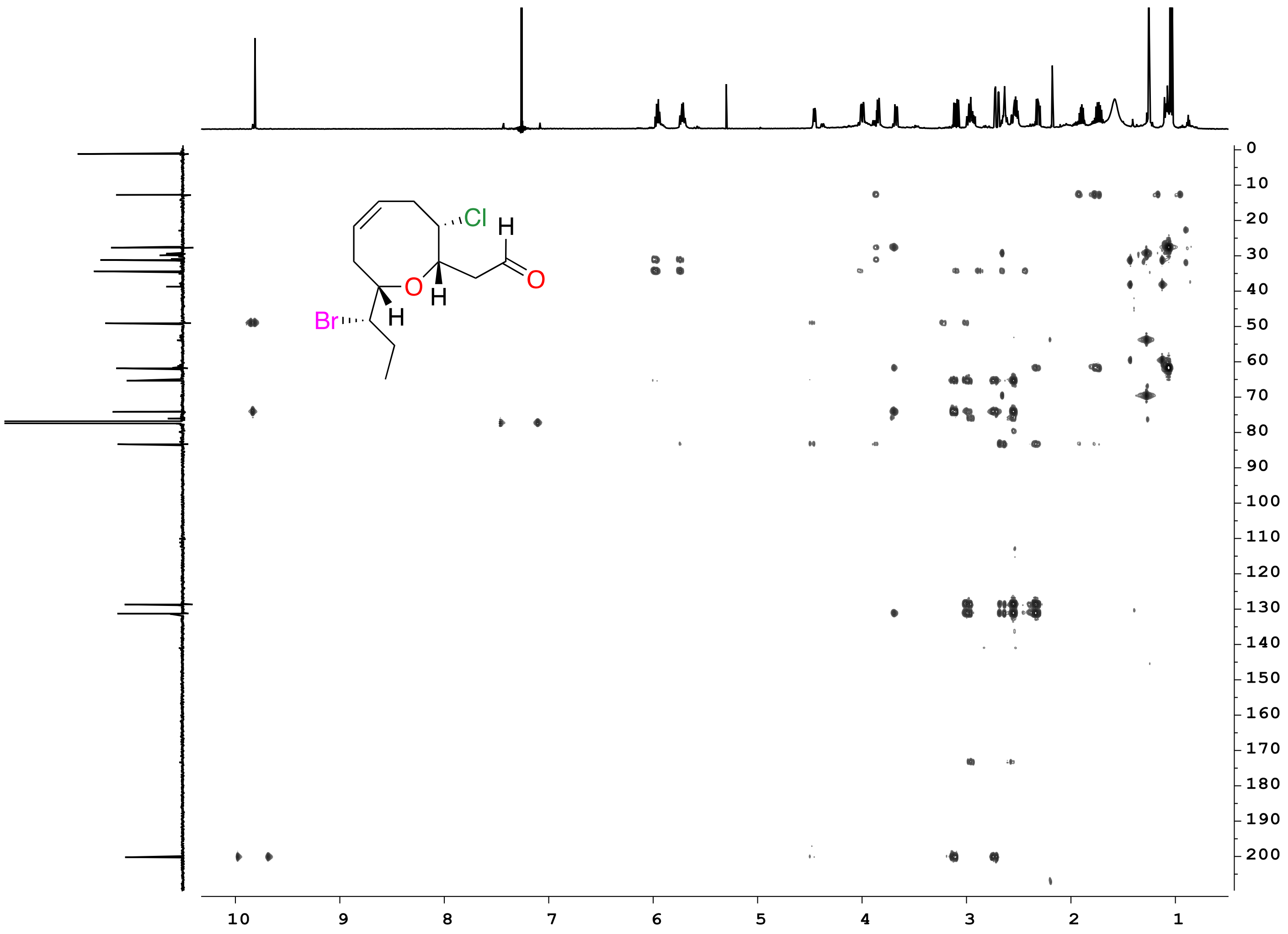

Figure S30. ROESY spectrum (600 MHz, $CDCl_3$) for pinnatifidehyde **6**.

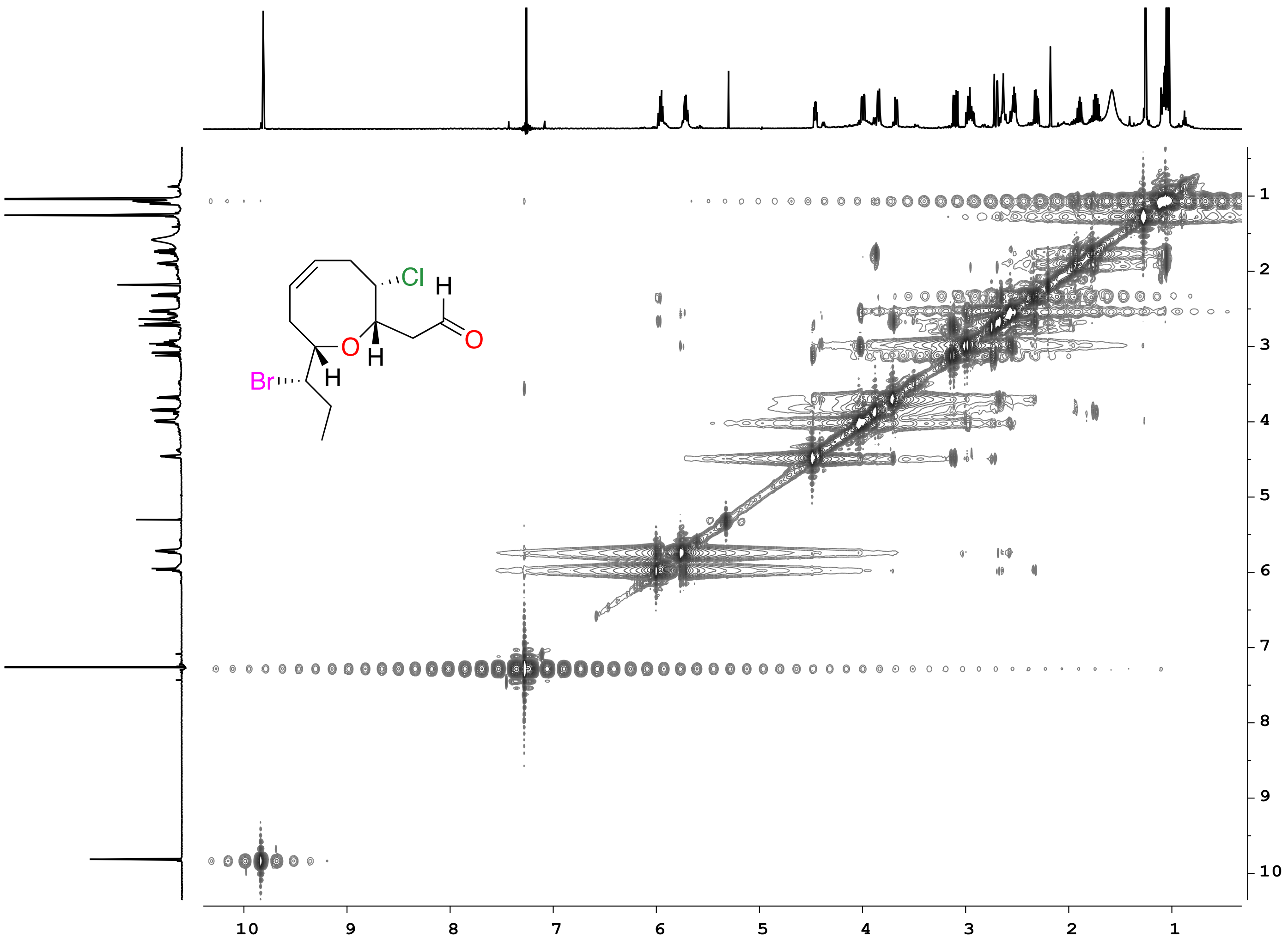

Figure S31. HRESIMS spectrum for pinnatifidehyde **6**.

Elemental Composition Report **Page 1**

Multiple Mass Analysis: 3 mass(es) processed
Tolerance = 5.0 PPM / DBE: min = -10.0, max = 1000.0
Element prediction: Off
Number of isotope peaks used for i-FIT = 2

Monoisotopic Mass, Even Electron Ions
1618 formula(e) evaluated with 13 results within limits (all results (up to 1000) for each mass)
Elements Used:
C: 0-60 H: 0-120 O: 0-3 Na: 0-1 35Cl: 0-1 37Cl: 0-1 79Br: 0-1 81Br: 0-1

ESI-318 Olivia M (Omf-17b) 137 (5.984) 1: TOF MS ES+ 2.07e+002

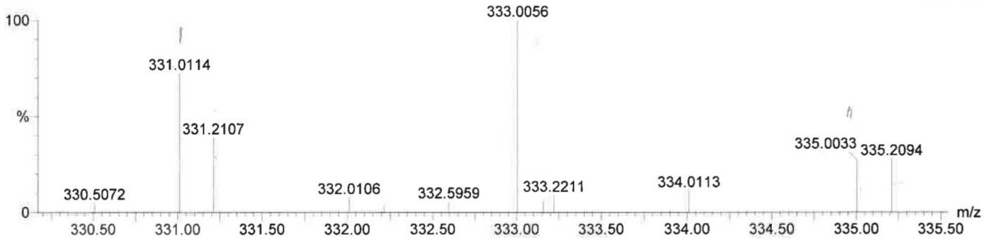

Mass	RA	Calc. Mass	mDa	PPM	DBE	i-FIT	Formula
Minimum:	30.00				-10.0		
Maximum:	100.00		100.0	5.0	1000.0		
331.0114	72.33	331.0107	0.7	2.1	12.5	4.5	C18 H11 O2 35Cl 37Cl
		331.0122	-0.8	-2.4	14.5	6.4	C20 H12 79Br
		331.0104	1.0	3.0	18.5	8.5	C22 H7 Na 37Cl
		331.0101	1.3	3.9	6.5	2.1	C15 H17 O 37Cl 81Br
		331.0100	1.4	4.2	5.5	1.5	C14 H17 O2 35Cl 79Br
		331.0129	-1.5	-4.5	21.5	10.7	C24 H6 37Cl
		331.0098	1.6	4.8	11.5	4.5	C18 H13 Na 79Br
331.2107	38.93	---					
333.0056	100.00	333.0056	0.0	0.0	2.5	0.5	C12 H18 O2 Na 35Cl 81Br
		333.0052	0.4	1.2	16.5	9.4	C21 H9 35Cl 37Cl
		333.0047	0.9	2.7	2.5	0.5	C12 H18 O2 Na 37Cl 79Br
		333.0046	1.0	3.0	9.5	4.4	C17 H15 35Cl 79Br
		333.0044	1.2	3.6	-9.5	2.3	C5 H28 O3 37Cl 79Br 81Br
		333.0071	-1.5	-4.5	5.5	1.7	C14 H17 O2 37Cl 79Br

Table S1. Calculated *vs* experimental RMSD for compounds **3** and **4**.

**Steroisomers**	RMSD (**3**)	RMSD (**4**)
3*R*,4*R*,6*R*,7*R*,12*R*,13*S*	2.3	2.3
3*R*,4*R*,6*R*,7*R*,12*S*,13*S*	3.8	3.8
3*R*,4*R*,6*R*,7*S*,12*R*,13*S*	3.0	2.7
3*R*,4*R*,6*R*,7*S*,12*S*,13*S*	2.7	2.6
3*R*,4*R*,6*S*,7*R*,12*R*,13*S*	2.6	2.7
3*R*,4*R*,6*S*,7*R*,12*S*,13*S*	3.0	2.7
3*R*,4*R*,6*S*,7*S*,12*R*,13*S*	2.4	2.5
3*R*,4*R*,6*S*,7*S*,12*S*,13*S*	2.2	2.2
3*R*,4*S*,6*R*,7*R*,12*R*,13*S*	2.2	2.0
3*R*,4*S*,6*R*,7*R*,12*S*,13*S*	3.6	3.5
3*R*,4*S*,6*R*,7*S*,12*R*,13*S*	3.7	3.4
3*R*,4*S*,6*R*,7*S*,12*S*,13*S*	2.5	2.3
3*R*,4*S*,6*S*,7*R*,12*R*,13*S*	2.2	2.2
3*R*,4*S*,6*S*,7*R*,12*S*,13*S*	2.4	2.3
3*R*,4*S*,6*S*,7*S*,12*R*,13*S*	3.3	3.4
3*R*,4*S*,6*S*,7*S*,12*S*,13*S*	1.6	1.6
3*S*,4*R*,6*R*,7*R*,12*R*,13*S*	2.0	2.0
3*S*,4*R*,6*R*,7*R*,12*S*,13*S*	3.5	3.5
3*S*,4*R*,6*R*,7*S*,12*R*,13*S*	3.0	2.7
3*S*,4*R*,6*R*,7*S*,12*S*,13*S*	3.1	3.1
3*S*,4*R*,6*S*,7*R*,12*R*,13*S*	2.5	2.5
3*S*,4*R*,6*S*,7*R*,12*S*,13*S*	2.7	2.4
3*S*,4*R*,6*S*,7*S*,12*R*,13*S*	2.2	2.3
3*S*,4*R*,6*S*,7*S*,12*S*,13*S*	1.9	1.8
3*S*,4*S*,6*R*,7*R*,12*R*,13*S*	2.3	2.3
3*S*,4*S*,6*R*,7*R*,12*S*,13*S*	3.8	3.9
3*S*,4*S*,6*R*,7*S*,12*R*,13*S*	3.3	3.0
3*S*,4*S*,6*R*,7*S*,12*S*,13*S*	2.4	2.4
3*S*,4*S*,6*S*,7*R*,12*R*,13*S*	2.9	3.0
3*S*,4*S*,6*S*,7*R*,12*S*,13*S*	2.9	2.8
3*S*,4*S*,6*S*,7*S*,12*R*,13*S*	2.9	3.0
3*S*,4*S*,6*S*,7*S*,12*S*,13*S*	2.2	2.3

Table S2. Scaled ^{13}C and ^{1}H chemical shifts for the two studied diastereoisomers on B3LYP-D3 in gas phase.

	3S,4R,6S,7S,12S,13S		***3R,4S,6S,7S,12S,13S***	
**#**	**δ_H**	**δ_C**	**δ_H**	**δ_C**
**1**	-	68.4	-	68.3
**2**	-	71.2	-	70.8
**3**	3.18	43.6	3.24	44.0
**4**	3.04	55.4	3.07	563
**5**	2.19<br>1.84	32.9	1.69<br>2.11	33.9
**6**	4.14	81.1	4.16	80.9
**7**	4.15	74.4	3.90	74.8
**8**	3.08<br>2.37	35.8	3.08<br>2.35	35.9
**9**	5.83	128.8	5.78	128.6
**10**	5.96	131.5	5.96	131.9
**11**	2.59<br>2.37	29.7	2.55<br>2.51	28.6
**12**	3.58	85.7	3.70	85.0
**13**	4.15	-	4.19	-
**14**	1.76<br>2.23	28.3	1.75<br>2.34	27.7
**15**	1.13	12.2	1.20	12.3

Figure S32. Correlations plots for ^{13}C and ^{1}H calculated *vs* ^{13}C and ^{1}H experimental data for (3*R*,4*S*)-epoxy-pinnatifidenyne **3**.

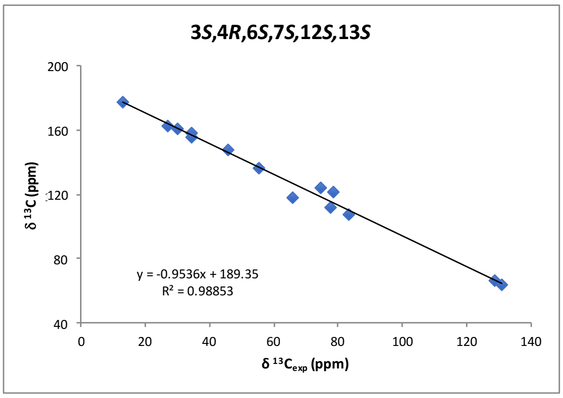

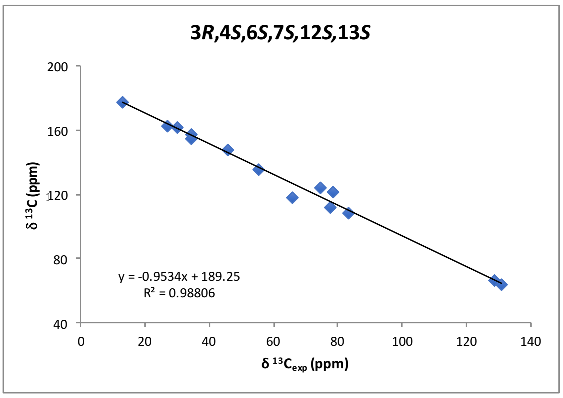

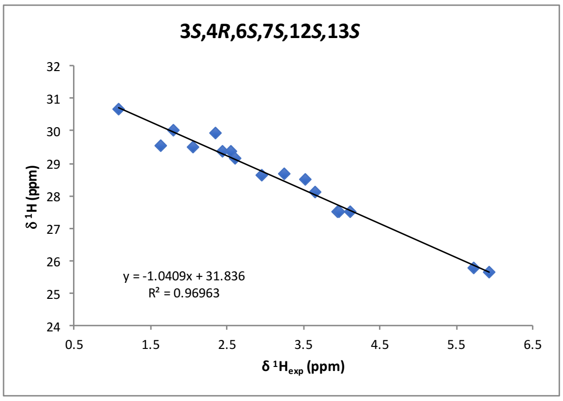

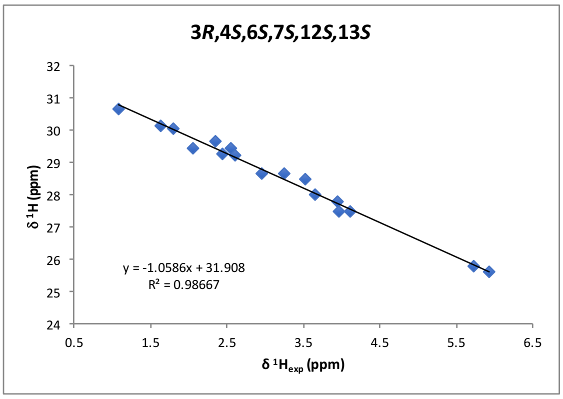

Figure S33. Correlations plots for ^{13}C and ^{1}H calculated *vs* ^{13}C and ^{1}H experimental data for (3*S*,4*R*)-epoxy-pinnatifidenyne **4**.

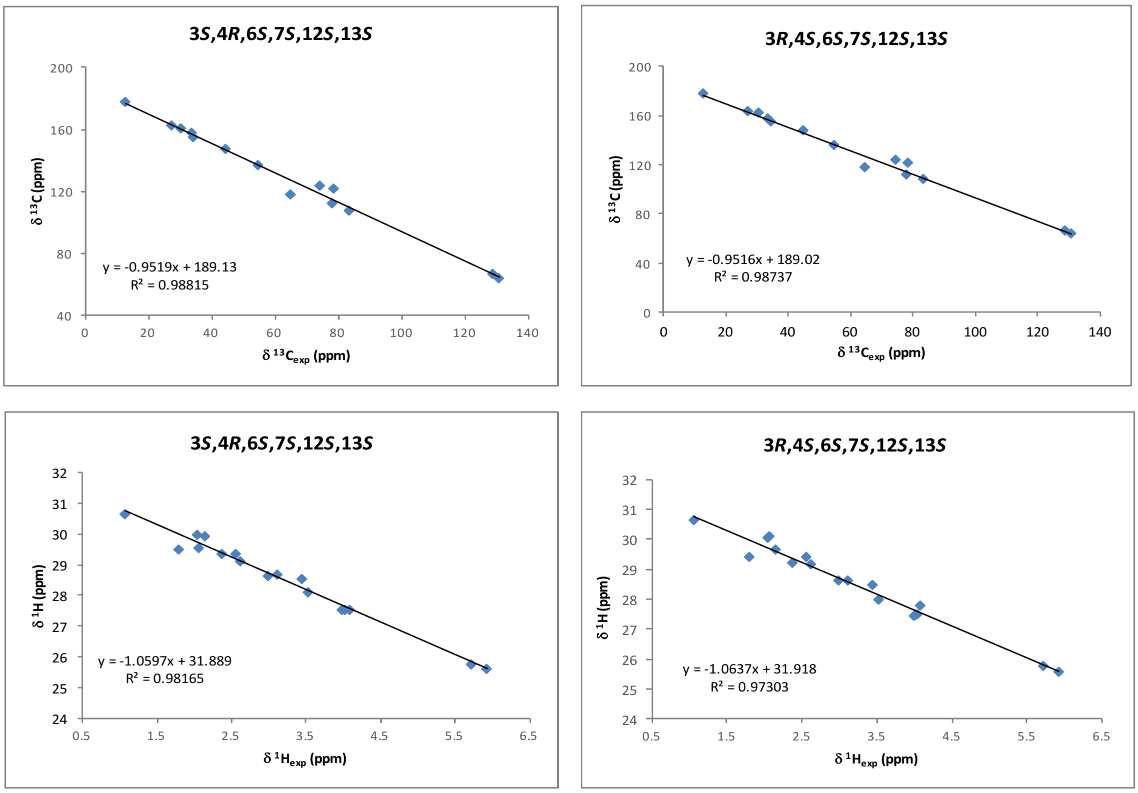

Table S3. Conformer 1 of 12 (RS_1).

Center Number		Coordinates			Center Number		Coordinates		
		X	Y	Z			X	Y	Z
2	C2	1,356258	0,224491	-0,149649	23	H23	1,818396	-0,295031	0,700887
3	C3	1,846167	1,676876	-0,046055	24	H24	2,93806	1,662938	0,035675
4	C4	1,206311	2,434528	1,131132	25	H25	0,141494	2,538087	0,903337
5	C5	1,402677	1,712816	2,439534	26	H26	1,64049	3,438288	1,170334
6	C6	0,550262	0,813415	2,947444	27	H27	2,330932	1,911787	2,973563
7	C7	-0,75943	0,392274	2,324352	28	H28	0,819951	0,319296	3,879849
8	C8	-0,592345	-0,540622	1,105381	29	H29	-1,31953	1,27295	1,990728
9	C9	1,777682	-0,513684	-1,423249	30	H30	-1,372681	-0,122564	3,069452
10	C10	1,350529	-1,95764	-1,33542	31	H31	2,864702	-0,453895	-1,540056
11	C11	2,213051	-3,095844	-1,723332	32	H32	1,312374	-0,045945	-2,296498
12	C12	3,539557	-2,905793	-2,258793	33	H33	5,613268	-2,628356	-3,148562
13	C13	4,642048	-2,760578	-2,732465	34	H34	-1,687026	-1,743721	-0,29668
15	H15	0,273148	-2,122568	-1,397724	35	H35	-3,347934	0,355857	1,184269
16	H16	1,723442	-4,021176	-2,033209	36	H36	-2,496812	0,669073	-0,32956
18	C18	-1,912239	-1,129828	0,577197	37	H37	-3,843753	-1,133817	-1,467677
19	C19	-2,997967	-0,112657	0,258404	38	H38	-4,699466	-1,452327	0,050156
20	C20	-4,179391	-0,682598	-0,527211	39	H39	-4,897049	0,106938	-0,769726
22	H22	0,077085	-1,373725	1,358867					

Table S4. Conformer 2 of 12 (RS_2).

Center Number		Coordinates			Center Number		Coordinates		
		X	Y	Z			X	Y	Z
2	C2	1,362858	0,230162	-0,179077	23	H23	1,821408	-0,293265	0,671404
3	C3	1,854694	1,681152	-0,067842	24	H24	2,947021	1,665095	0,010027
4	C4	1,221533	2,431804	1,117393	25	H25	0,155771	2,540323	0,895598
5	C5	1,420741	1,701442	2,41987	26	H26	1,658849	3,434047	1,160847
6	C6	0,569507	0,798682	2,9235	27	H27	2,35068	1,895517	2,952661
7	C7	-0,742475	0,382197	2,302539	28	H28	0,842179	0,29805	3,851508
8	C8	-0,585179	-0,536854	1,072464	29	H29	-1,308299	1,265904	1,987179
9	C9	1,78898	-0,505632	-1,453104	30	H30	-1,347545	-0,146659	3,045258
10	C10	1,332197	-1,941381	-1,38402	31	H31	2,879275	-0,467949	-1,548524
11	C11	2,188318	-3,096427	-1,734507	32	H32	1,348654	-0,023436	-2,331424
12	C12	3,541395	-2,935938	-2,209075	33	H33	5,660873	-2,707457	-2,998477
13	C13	4,667971	-2,8164	-2,629798	34	H34	-1,712866	-1,713418	-0,327146
15	H15	0,255624	-2,086374	-1,490717	35	H35	-3,965953	-0,804963	0,090447
16	H16	1,693441	-4,012	-2,064437	36	H36	-3,353844	0,279879	1,324554
18	C18	-1,916155	-1,140145	0,578648	37	H37	-2,613233	0,393852	-1,6726
19	C19	-3,099228	-0,198925	0,373057	38	H38	-3,743777	1,474975	-0,837302
20	C20	-2,846083	0,862074	-0,710883	39	H39	-2,007285	1,513555	-0,460594
22	H22	0,090089	-1,367772	1,31567					

Table S5. Conformer 3 of 12 (RS_7).

Center Number		Coordinates			Center Number		Coordinates		
		X	Y	Z			X	Y	Z
2	C2	1,372806	0,065053	-0,052312	23	H23	1,791594	-0,419661	0,839934
3	C3	1,899555	1,511021	-0,002775	24	H24	2,984042	1,479457	0,143799
4	C4	1,220054	2,361754	1,083902	25	H25	0,176075	2,491045	0,785542
5	C5	1,310103	1,723182	2,446645	26	H26	1,688657	3,351002	1,088481
6	C6	0,396201	0,890906	2,961066	27	H27	2,209773	1,931914	3,024214
7	C7	-0,885221	0,464438	2,285302	28	H28	0,587096	0,460651	3,943253
8	C8	-0,656876	-0,560302	1,15319	29	H29	-1,39324	1,330543	1,846914
9	C9	1,78806	-0,754727	-1,281855	30	H30	-1,564916	0,0275	3,022579
10	C10	3,277181	-0,740639	-1,542639	31	H31	1,256078	-0,371436	-2,157893
11	C11	4,216939	-1,595605	-0,771021	32	H32	1,46898	-1,788581	-1,12064
12	C12	3,747272	-2,472509	0,277509	33	H33	3,02837	-3,832788	1,952136
13	C13	3,365586	-3,191997	1,170886	34	H34	-1,665058	-1,860698	-0,224075
15	H15	3,680787	0,191509	-1,943244	35	H35	-3,391567	0,363696	0,982649
16	H16	5,248578	-1,257214	-0,656258	36	H36	-2,448576	0,547371	-0,496235
18	C18	-1,943565	-1,180269	0,582703	37	H37	-4,699875	-1,51704	-0,088341
19	C19	-2,993534	-0,181378	0,119894	38	H38	-4,826271	-0,023395	-1,032968
20	C20	-4,134674	-0,798753	-0,689192	39	H39	-3,751758	-1,322464	-1,572196
22	H22	-0,015496	-1,375621	1,515397					

Table S6. Conformer 4 of 12 (RS_11).

Center Number		Coordinates			Center Number		Coordinates		
		X	Y	Z			X	Y	Z
2	C2	1,390582	0,054795	-0,062719	23	H23	1,77462	-0,448905	0,836303
3	C3	1,966832	1,484455	0,003727	24	H24	3,046066	1,41317	0,177406
4	C4	1,292512	2,353084	1,075403	25	H25	0,255443	2,504812	0,761942
5	C5	1,351847	1,711905	2,436511	26	H26	1,786076	3,330296	1,085978
6	C6	0,408695	0,906496	2,939453	27	H27	2,252271	1,893314	3,022538
7	C7	-0,875689	0,522429	2,246295	28	H28	0,575673	0,47245	3,924627
8	C8	-0,669351	-0,49683	1,103885	29	H29	-1,368647	1,399872	1,815611
9	C9	1,807095	-0,771912	-1,289043	30	H30	-1,573592	0,093147	2,974323
10	C10	3,30417	-0,865623	-1,473866	31	H31	1,348262	-0,334231	-2,180856
11	C11	4,130572	-1,809698	-0,676562	32	H32	1,408116	-1,783032	-1,170711
12	C12	3,535173	-2,67388	0,316713	33	H33	2,628272	-4,019105	1,907662
13	C13	3,049541	-3,383415	1,164633	34	H34	-2,456859	-1,637133	1,486485
15	H15	3,799448	0,04124	-1,826388	35	H35	-2,745073	-2,532034	-0,83072
16	H16	5,176954	-1,556541	-0,493762	36	H36	-1,299975	-1,663059	-1,349403
18	C18	-1,956662	-1,178372	0,629187	37	H37	0,123136	-3,108247	0,159088
19	C19	-1,761288	-2,181308	-0,5036	38	H38	-1,332652	-3,887212	0,800163
20	C20	-0,908293	-3,387047	-0,078528	39	H39	-0,867728	-4,120112	-0,890037
22	H22	-0,042755	-1,318627	1,491584					

Table S7. Conformer 5 of 12 (RS_12).

Center Number		Coordinates			Center Number		Coordinates		
		X	Y	Z			X	Y	Z
2	C2	1,377524	0,059978	-0,061933	23	H23	1,788541	-0,429875	0,831345
3	C3	1,90634	1,504753	-0,002903	24	H24	2,990418	1,470349	0,145732
4	C4	1,227476	2,353251	1,085781	25	H25	0,185674	2,492725	0,784163
5	C5	1,306026	1,70756	2,445611	26	H26	1,703013	3,339087	1,097759
6	C6	0,384808	0,877267	2,949989	27	H27	2,203371	1,908359	3,029519
7	C7	-0,893989	0,459763	2,263702	28	H28	0,567244	0,441566	3,931301
8	C8	-0,663691	-0,558889	1,127348	29	H29	-1,399147	1,330377	1,83104
9	C9	1,799758	-0,75388	-1,293178	30	H30	-1,577438	0,016615	2,994413
10	C10	3,289857	-0,736796	-1,548295	31	H31	1,270764	-0,366782	-2,169334
11	C11	4,227443	-1,593563	-0,776345	32	H32	1,481252	-1,78871	-1,137991
12	C12	3,753933	-2,474466	0,266911	33	H33	3,02671	-3,840988	1,932699
13	C13	3,368591	-3,197423	1,15574	34	H34	-1,68986	-1,830139	-0,264834
15	H15	3,693981	0,19743	-1,943419	35	H35	-3,966041	-0,879685	-0,069707
16	H16	5,258193	-1,254443	-0,656211	36	H36	-3,42468	0,296287	1,110867
18	C18	-1,957309	-1,190428	0,577733	37	H37	-3,666794	1,292995	-1,184949
19	C19	-3,111838	-0,259142	0,219971	38	H38	-1,973829	1,399907	-0,659882
20	C20	-2,776955	0,710541	-0,925645	39	H39	-2,450879	0,169199	-1,819354
22	H22	-0,021498	-1,372536	1,490993					

Table S8. Conformer 6 of 12 (RS_12).

Center Number		Coordinates			Center Number		Coordinates		
		X	Y	Z			X	Y	Z
2	C2	1,359151	0,110221	-0,105043	23	H23	1,639856	3,385589	1,072554
3	C3	1,857889	1,565315	-0,04928	24	H24	2,23504	1,936838	2,967823
4	C4	1,188017	2,388688	1,062688	25	H25	0,659496	0,414891	3,891978
5	C5	1,323442	1,726781	2,410107	26	H26	-1,379694	1,306276	1,860251
6	C6	0,437046	0,866932	2,926447	27	H27	-1,511479	-0,020571	3,014687
7	C7	-0,853437	0,43744	2,270516	28	H28	0,003876	-1,386486	1,451238
8	C8	-0,644701	-0,5658	1,115098	29	H29	1,284864	-0,307656	-2,222271
9	C9	1,80021	-0,693132	-1,337037	30	H30	1,486327	-1,730677	-1,188104
10	C10	3,293757	-0,62362	-1,547423	31	H31	3,660624	0,297466	-2,005719
11	C11	4,150502	-1,823822	-1,708193	32	H32	5,085333	-1,709734	-2,260401
12	C12	3,605019	-3,159997	-1,715578	33	H33	2,768504	-5,273158	-1,765395
13	C13	3,161686	-4,283547	-1,744018	34	H34	-1,687807	-1,834242	-0,267549
16	C16	-1,944771	-1,176002	0,564387	35	H35	-3,377558	0,356934	1,048411
17	C17	-3,010917	-0,168764	0,160194	36	H36	-2,487993	0,573508	-0,45893
18	C18	-4,182042	-0,76828	-0,618647	37	H37	-3,833801	-1,273251	-1,526515
20	H20	1,799939	-0,37032	0,778064	38	H38	-4,724798	-1,498903	-0,011957
21	H21	2,945311	1,552538	0,071317	39	H39	-4,885254	0,014858	-0,918637
22	H22	0,134918	2,503409	0,791398					

Table S9. Conformer 7 of 12 (SR_1).

Center Number		Coordinates			Center Number		Coordinates		
		X	Y	Z			X	Y	Z
2	C2	1,423953	0,299663	-0,156669	23	H23	1,574963	3,581268	1,020753
3	C3	1,873694	1,77118	-0,09606	24	H24	2,277649	2,190017	2,921685
4	C4	1,179942	2,560654	1,027883	25	H25	0,793126	0,610718	3,894907
5	C5	1,370965	1,924656	2,379356	26	H26	-1,32801	1,354946	1,891388
6	C6	0,534688	1,032257	2,924388	27	H27	-1,351462	0,025458	3,052716
7	C7	-0,739525	0,522925	2,29468	28	H28	0,24139	-1,227288	1,459729
8	C8	-0,48857	-0,470766	1,140847	29	H29	2,975792	-0,580681	-1,36988
9	C9	1,88732	-0,465213	-1,402721	30	H30	1,630914	0,088126	-2,310691
10	C10	1,245898	-1,832944	-1,471738	31	H31	0,197422	-1,832962	-1,771924
11	C11	1,685245	-2,975043	-0,632723	32	H32	0,947466	-3,742006	-0,387792
12	C12	2,762031	-2,844773	0,320054	33	H33	4,417043	-2,649569	1,867308
13	C13	3,639746	-2,737843	1,145043	34	H34	-1,4641	-1,862818	-0,170364
16	C16	-1,747982	-1,205896	0,653293	35	H35	-3,296335	0,205931	1,148369
17	C17	-2,913687	-0,311171	0,261869	36	H36	-2,47739	0,45516	-0,394574
18	C18	-4,04694	-1,038628	-0,462579	37	H37	-3,675086	-1,550422	-1,357152
20	H20	1,890763	-0,173067	0,719097	38	H38	-4,509881	-1,787021	0,18668
21	H21	2,962961	1,786366	0,018454	39	H39	-4,820546	-0,331209	-0,77577
22	H22	0,117552	2,610883	0,771239					

Table S10. Conformer 8 of 12 (SR_10).

Center Number		Coordinates			Center Number		Coordinates		
		X	Y	Z			X	Y	Z
2	C2	1,364764	0,10005	-0,108694	23	H23	1,68717	3,368483	1,077735
3	C3	1,881189	1,548088	-0,047204	24	H24	2,246176	1,914556	2,977149
4	C4	1,219143	2,379221	1,064246	25	H25	0,645005	0,415569	3,891939
5	C5	1,336551	1,715152	2,412454	26	H26	-1,375337	1,328568	1,854901
6	C6	0,435348	0,867709	2,923704	27	H27	-1,519579	-0,005826	2,999229
7	C7	-0,856179	0,452998	2,259693	28	H28	-0,008396	-1,369964	1,440334
8	C8	-0,653625	-0,547969	1,10205	29	H29	1,276866	-0,321762	-2,223636
9	C9	1,799323	-0,705161	-1,341841	30	H30	1,487338	-1,742468	-1,187711
10	C10	3,29105	-0,633616	-1,565496	31	H31	3,649375	0,277902	-2,048746
11	C11	4,152993	-1,833181	-1,702418	32	H32	5,08294	-1,728584	-2,264445
12	C12	3,615479	-3,172103	-1,667779	33	H33	2,791172	-5,290574	-1,646132
13	C13	3,178578	-4,298462	-1,658759	34	H34	-1,722381	-1,771188	-0,301387
16	C16	-1,963495	-1,164223	0,572672	35	H35	-3,993324	-0,835801	0,002808
17	C17	-3,131638	-0,22418	0,289008	36	H36	-3,416319	0,293744	1,211268
18	C18	-2,836781	0,792334	-0,826491	37	H37	-3,733451	1,387707	-1,026118
20	H20	1,795451	-0,38847	0,774735	38	H38	-2,020847	1,46724	-0,562982
21	H21	2,967898	1,52097	0,076553	39	H39	-2,547469	0,288479	-1,754074
22	H22	0,169208	2,512939	0,789476					

Table S11. Conformer 9 of 12 (SR_9).

Center Number		Coordinates			Center Number		Coordinates		
		X	Y	Z			X	Y	Z
2	C2	1,40512	0,116423	-0,167171	23	H23	1,715929	3,398943	0,986445
3	C3	1,920551	1,568189	-0,120075	24	H24	2,337183	1,957086	2,877621
4	C4	1,262078	2,402589	0,987862	25	H25	0,771788	0,441249	3,830102
5	C5	1,416751	1,746192	2,334446	26	H26	-1,30864	1,338105	1,842258
6	C6	0,536788	0,88917	2,865301	27	H27	-1,410535	0,005035	2,983746
7	C7	-0,765624	0,469573	2,228579	28	H28	0,054689	-1,349384	1,401201
8	C8	-0,594176	-0,524518	1,057395	29	H29	1,343673	-0,297738	-2,287995
9	C9	1,839779	-0,695594	-1,397115	30	H30	1,50051	-1,7258	-1,256326
10	C10	3,336575	-0,66489	-1,589807	31	H31	3,73227	0,238647	-2,059379
11	C11	4,160955	-1,89193	-1,716576	32	H32	5,105663	-1,818211	-2,259454
12	C12	3,574738	-3,210288	-1,701655	33	H33	2,672654	-5,295158	-1,720698
13	C13	3,095733	-4,318165	-1,712881	34	H34	-2,344278	-1,70153	1,493755
16	C16	-1,897564	-1,205098	0,627908	35	H35	-2,758555	-2,505644	-0,838736
17	C17	-1,759819	-2,159768	-0,554015	36	H36	-1,351792	-1,602865	-1,402676
18	C18	-0,8758	-3,373871	-0,230419	37	H37	-1,242867	-3,914554	0,649955
20	H20	1,843682	-0,366192	0,717221	38	H38	-0,878068	-4,071854	-1,073532
21	H21	3,008377	1,543037	0,000254	39	H39	0,165504	-3,094576	-0,043278
22	H22	0,205527	2,515782	0,727448					

Table S12. Conformer 10 of 12 (RS_2).

Center Number		Coordinates			Center Number		Coordinates		
		X	Y	Z			X	Y	Z
2	C2	1,419332	0,279088	-0,170584	23	H23	1,548584	3,566262	0,993369
3	C3	1,856343	1,755851	-0,11738	24	H24	2,256613	2,182923	2,897561
4	C4	1,158034	2,543806	1,003337	25	H25	0,781526	0,596294	3,871504
5	C5	1,35175	1,911697	2,355052	26	H26	-1,347267	1,32572	1,87614
6	C6	0,520797	1,014559	2,900117	27	H27	-1,352316	-0,012009	3,029808
7	C7	-0,749302	0,496203	2,270965	28	H28	0,240129	-1,242254	1,436588
8	C8	-0,497543	-0,494628	1,11542	29	H29	2,992054	-0,590731	-1,362574
9	C9	1,901966	-0,49534	-1,40385	30	H30	1,639005	0,039459	-2,321004
10	C10	1,289732	-1,877041	-1,455825	31	H31	0,241099	-1,903771	-1,755277
11	C11	1,751834	-2,998195	-0,601791	32	H32	1,028648	-3,776031	-0,347516
12	C12	2,823152	-2,833277	0,350807	33	H33	4,468392	-2,582694	1,899351
13	C13	3,695624	-2,697146	1,176275	34	H34	-1,490767	-1,886826	-0,18606
16	C16	-1,761237	-1,251712	0,658803	35	H35	-3,824221	-1,139784	0,12248
17	C17	-3,015393	-0,439495	0,354861	36	H36	-3,324467	0,10555	1,253447
18	C18	-2,827846	0,530119	-0,824207	37	H37	-3,766457	1,059802	-1,013115
20	H20	1,879393	-0,181854	0,715951	38	H38	-2,043083	1,26309	-0,630821
21	H21	2,945764	1,781126	-0,002342	39	H39	-2,548195	-0,008829	-1,735307
22	H22	0,094751	2,588231	0,747202					

Table S13. Conformer 11 of 12 (SR_6).

Center Number		Coordinates			Center Number		Coordinates		
		X	Y	Z			X	Y	Z
2	C2	1,39666	0,087493	-0,130463	23	H23	1,743147	3,353468	1,053318
3	C3	1,935103	1,528132	-0,064219	24	H24	2,321889	1,89893	2,947269
4	C4	1,276158	2,363601	1,044382	25	H25	0,72638	0,404435	3,878657
5	C5	1,405973	1,701374	2,391772	26	H26	-1,322413	1,32882	1,871982
6	C6	0,50817	0,856112	2,911785	27	H27	-1,450744	-0,009243	3,003907
7	C7	-0,792572	0,453294	2,259358	28	H28	0,018186	-1,370887	1,423864
8	C8	-0,618833	-0,533838	1,083501	29	H29	1,294986	-0,310133	-2,249546
9	C9	1,814031	-0,712088	-1,373799	30	H30	1,485802	-1,746156	-1,232447
10	C10	3,305285	-0,661705	-1,601106	31	H31	3,679559	0,259142	-2,053758
11	C11	4,144074	-1,871409	-1,784749	32	H32	5,073693	-1,764365	-2,347228
12	C12	3,582792	-3,201026	-1,798247	33	H33	2,730801	-5,307017	-1,86396
13	C13	3,130378	-4,320329	-1,834189	34	H34	-2,387358	-1,694241	1,493913
16	C16	-1,918643	-1,208213	0,634104	35	H35	-1,394976	-1,620558	-1,399372
17	C17	-1,723891	-2,183007	-0,521293	36	H36	-0,881325	-2,831638	-0,235248
18	C18	-2,944871	-3,047625	-0,839043	37	H37	-2,715339	-3,755605	-1,64149
20	H20	1,829508	-0,413181	0,745825	38	H38	-3,2629	-3,624552	0,037179
21	H21	3,020427	1,483983	0,068797	39	H39	-3,789099	-2,432341	-1,161274
22	H22	0,224535	2,491771	0,772943					

Table S14. Conformer 12 of 12 (SR_12).

Center Number		Coordinates			Center Number		Coordinates		
		X	Y	Z			X	Y	Z
2	C2	1,384409	0,053347	-0,080571	23	H23	1,617222	3,346966	1,051603
3	C3	1,881041	1,510223	-0,03508	24	H24	2,164624	1,932832	2,980968
4	C4	1,1718	2,347122	1,04158	25	H25	0,59203	0,413385	3,887911
5	C5	1,271136	1,706456	2,400428	26	H26	-1,44379	1,217684	1,808514
6	C6	0,382401	0,845298	2,910056	27	H27	-1,534217	-0,072058	3,007616
7	C7	-0,891567	0,376821	2,245775	28	H28	0,02407	-1,442571	1,465316
8	C8	-0,63205	-0,64062	1,098624	29	H29	1,315213	-0,38289	-2,192084
9	C9	1,829439	-0,761693	-1,303647	30	H30	1,506498	-1,794745	-1,150355
10	C10	3,323019	-0,690076	-1,509752	31	H31	3,686766	0,209259	-2,011517
11	C11	4,189342	-1,891004	-1,601085	32	H32	5,126707	-1,801731	-2,153935
12	C12	3,649636	-3,227447	-1,537048	33	H33	2,805002	-5,335674	-1,473525
13	C13	3,205248	-4,34945	-1,505154	34	H34	-2,457551	-0,512802	-0,027937
16	C16	-1,933697	-1,256029	0,576549	35	H35	-3,296035	-1,066699	2,198744
17	C17	-2,86986	-1,88798	1,605722	36	H36	-3,71237	-2,337003	1,069785
18	C18	-2,229932	-2,923516	2,535273	37	H37	-1,433877	-2,488276	3,148603
20	H20	1,82024	-0,418946	0,809895	38	H38	-2,979855	-3,340126	3,214606
21	H21	2,964561	1,50159	0,116822	39	H39	-1,798931	-3,746725	1,95892
22	H22	0,126423	2,448908	0,736976					

Figure S34. DP4 app for (3*R*,4*S*)-epoxy-pinnatifidenyne **3**.

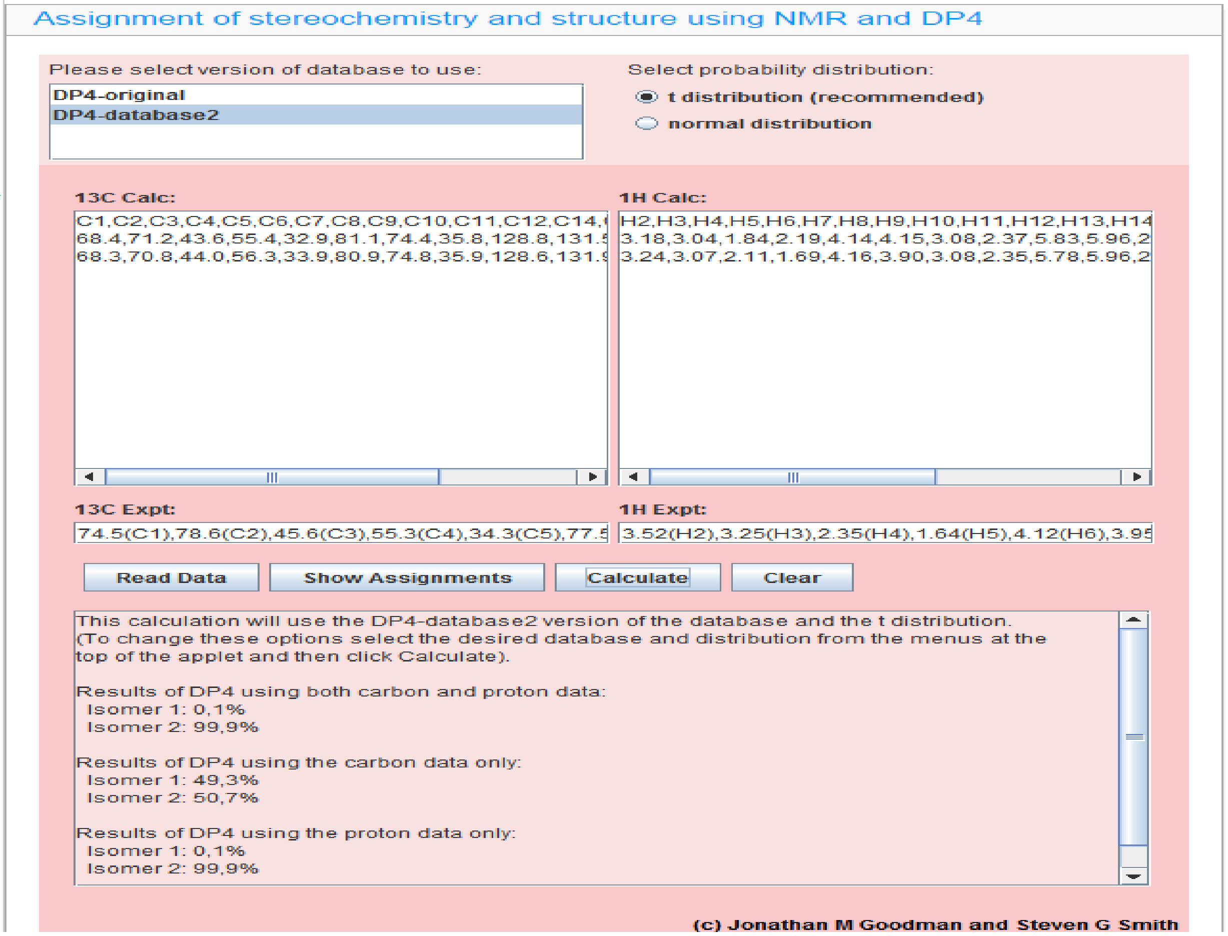

Table S15. Antiproliferative activity against human cancer cell lines.

	GI_{50} (μM)					
**Compound**	**A549**	**HBL-100**	**HeLa**	**SW1573**	**T-47D**	**WiDr**
**3**	>50	>50	>50	>50	>50	>50
**4**	33 ± 17	>50	45 ± 6.7	>50	>50	>50
**5**	35 ± 4.9	>50	36 ± 14	13 ± 0.6	48 ± 3.8	>50
**6**	>50	>50	>50	>50	>50	>50
